# A Compendium of Canine Normal Tissue Gene Expression

**DOI:** 10.1371/journal.pone.0017107

**Published:** 2011-05-31

**Authors:** Joseph Briggs, Melissa Paoloni, Qing-Rong Chen, Xinyu Wen, Javed Khan, Chand Khanna

**Affiliations:** 1 Tumor and Metastasis Biology Section, Pediatric Oncology Branch, Center for Cancer Research, National Cancer Institute, National Institutes of Health, Bethesda, Maryland, United States of America; 2 Comparative Oncology Program, Center for Cancer Research, National Cancer Institute, National Institutes of Health, Bethesda, Maryland, United States of America; 3 Oncogenomics Section, Pediatric Oncology Branch, Center for Cancer Research, National Cancer Institute, National Institutes of Health, Bethesda, Maryland, United States of America; Health Canada, Canada

## Abstract

**Background:**

Our understanding of disease is increasingly informed by changes in gene expression between normal and abnormal tissues. The release of the canine genome sequence in 2005 provided an opportunity to better understand human health and disease using the dog as clinically relevant model. Accordingly, we now present the first genome-wide, canine normal tissue gene expression compendium with corresponding human cross-species analysis.

**Methodology/Principal Findings:**

The Affymetrix platform was utilized to catalogue gene expression signatures of 10 normal canine tissues including: liver, kidney, heart, lung, cerebrum, lymph node, spleen, jejunum, pancreas and skeletal muscle. The quality of the database was assessed in several ways. Organ defining gene sets were identified for each tissue and functional enrichment analysis revealed themes consistent with known physio-anatomic functions for each organ. In addition, a comparison of orthologous gene expression between matched canine and human normal tissues uncovered remarkable similarity. To demonstrate the utility of this dataset, novel canine gene annotations were established based on comparative analysis of dog and human tissue selective gene expression and manual curation of canine probeset mapping. Public access, using infrastructure identical to that currently in use for human normal tissues, has been established and allows for additional comparisons across species.

**Conclusions/Significance:**

These data advance our understanding of the canine genome through a comprehensive analysis of gene expression in a diverse set of tissues, contributing to improved functional annotation that has been lacking. Importantly, it will be used to inform future studies of disease in the dog as a model for human translational research and provides a novel resource to the community at large.

## Introduction

The opportunity to study health and disease in the dog (*Canis lupus familiaris*) has significantly expanded with the release of the first public draft of the canine genome. [1], [2], [3] This opportunity has been complemented by the development of high throughput technologies, such as expression and SNP microarrays, now commercially available for the dog [4], [5], [6]. Using these techniques and data, questions and hypotheses related to the health of dogs and their inclusion in biomedical research can now be articulated from a post-genomic perspective. [7], [8], [9] However, our ability to extend and refine our knowledge is limited due to the lack of a comprehensive functional assessment of canine gene expression in diverse sets of normal tissues. [10], [11] Rather than repeating this requisite step in new canine genomic studies, an efficient approach would be to provide researchers with an openly accessible set of validated expression profiles from canine normal tissues. A similar approach has been used for human normal organ gene expression data on both oligonucleotide and cDNA array platforms. [12], [13], [14], [15]. A major benefit of these human studies is that several datasets are publicly available through web-based interactive analytical tools. Based on the same rationale and using a similar approach, the availability of an online database of canine normal tissue gene expression profiles would serve as the foundation for *in silico* analysis of canine diseases thereby increasing the efficiency and eliminating redundancy. Since the dog represents a model organism for human disease, the development of such a database would also enable more rigorous comparative genomic analysis with gene expression data sets available for human, rat and murine tissues [2]. Such comparative studies would enable the identification of common gene regulatory regions as well as evolutionarily conserved gene expression networks providing a better understanding of organ functions in normal and diseased states.

To meet these needs and opportunities, the goal of this project was to develop a robust, publicly accessible gene expression profile database from ten normal canine organs using the Affymetrix Canine Version 2.0 GeneChip® platform. Tissues included: liver, kidney, heart, lung, cerebrum, spleen, lymph node, jejunum, pancreas, and skeletal muscle. The informative utility of the resultant expression data was assessed in several ways. Bioinformatic analysis revealed a large number of differentially expressed genes based on tissue type. This enabled the identification of gene expression profiles that were selective for each tissue. Indeed, hierarchical clustering and principle component analysis using these profiles demonstrated that organs grouped together based on shared function and structural composition. Consistent with these observations, analyses of tissue selective genes were suggestive of tissue origin, function and physiology. Importantly, analysis of canine and human orthologous gene expression in matched tissues revealed remarkable similarity between species. These normal tissue expression data and the demonstration of shared orthologous expression patterns in humans allowed redefinition of canine Affymetrix probesets not previously mapped to a known transcript. In the future, this data should aid in expanding and refining canine gene ontologies allowing a much more robust assessment of biological functions associated with co-regulated gene sets in each tissue.

This data is now publicly available for use through the establishment of a Canine Normal Tissue Database (ccr.cancer.gov/resources/cop). This database allows for gene specific queries of normal tissues in the dog as well as cross species comparison of gene expression between canine and human tissues. We anticipate this dataset will provide the foundation for more advanced study of disease in the dog and improve biomedical studies that utilize the dog as a model for translational research.

## Results

### Validation of an Informative Database of Normal Canine Tissues: Identification of Organ Selective Gene Expression Signatures

Previous studies have cataloged global gene expression patterns for normal tissues in pig, mouse, rat and human. [12], [15], [16], [17], [18], [19], [20], [21] The dog is an excellent model for many human diseases, however little is known about canine gene expression across diverse tissues. Therefore, ten organs from four dogs were harvested and RNA purified for analysis using Affymetrix Canine Version 2.0 arrays. This array consists of 42,860 canine probesets corresponding to over 18,000 mRNA/EST based transcripts and over 20,000 non-redundant predicted genes (www.affymetrix.com). [4]

**Figure 1:**
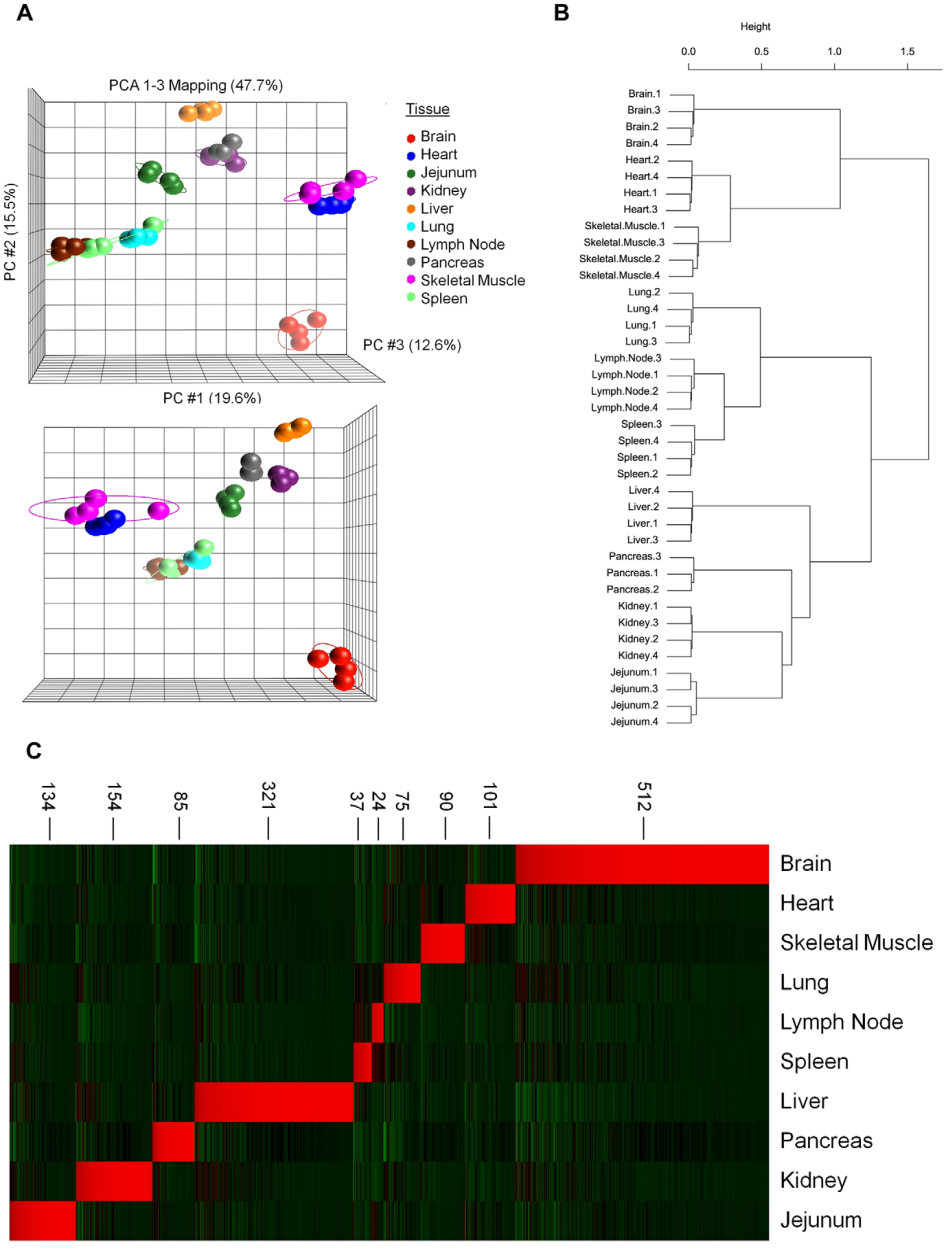
Principle component analysis and hierarchical clustering define relationships between canine normal tissues. mRNA expression for 39 samples from ten pathologically normal canine tissues were analyzed using the Affymetrix Canine Version 2.0 GeneChip®. Probesets differentially expressed in at least one tissue (as described in the Methods) were included in the analysis (23,070 probesets corresponding to 10,878 unique gene symbols). **A**. Samples were analyzed by principle component analysis (PCA) to characterize relationships between biological replicates for each tissue. Each sphere represents an individual sample, colored by tissue and ellipses correspond to two standard deviations of the tissue group mean. **B**. Hierarchical clustering of samples was conducted with distances calculated using Pearson correlation metrics and clusters joined using Ward linkage. Bootstrap re-sampling was conducted (1,000 iterations) in order to determine cluster stability. **C**. Heatmap demonstrating tissue selective gene expression. Following ANOVA to determine differential expression based on tissue type, results were filtered based on FDR = 0.001 as well as expression thresholds of greater than 10-fold expression over the mean of all other tissues and no greater than 3-fold over the mean in any other tissue. This final list of tissue selective probesets was rank ordered according to fold-expression with sample order determined by the previous bootstrapped hierarchical clustering. Red indicates upregulated and green represents downregulated relative to the mean expression in all tissues. Numbers next to the heatmap indicate the number of tissue selective probesets in a cluster.

A comparison of gene expression profiles for ten normal canine organs was undertaken using an ANOVA model to assess the informative value of this data set. Consistent with previous studies in humans, >50% of all canine probesets (23,070) demonstrated differential expression based on tissue type and this corresponds to 10,878 unique gene symbols. [15], [19], [20] To further validate the utility of these data and to characterize relationships between biological replicates, samples were analyzed by principle component analysis (PCA) (**Fig. 1A** **and Fig. S1**) and hierarchical clustering (HC) (**Fig. 1B**) using those probesets differentially expressed in at least one tissue. As shown in **Fig. 1A** samples grouped according to organ type with greater than 47% of the variability explained by the first three principle components. Multi-level bootstrap re-sampling was then conducted on hierarchical clustering results in order to determine the reproducibility of cluster assignment. As shown in **Fig. 1B**, replicate samples again grouped together according to organ type (>95% confidence at each branch point). Identical results were observed when using all probesets (data not shown). In addition, tissues with a common developmental origin and/or anatomical function grouped together. For example, mesoderm derived heart and skeletal muscle group together as do the functionally related immune organs lymph node and spleen.

A further assessment of the quality and informative value of the data was provided by defining contrasts as part of the ANOVA model. Probe sets that were differentially expressed in one tissue versus all others were identified following correction for multiple testing (FDR, 0.001). The number of differentially expressed probesets in each tissue, as well as the corresponding number of unique gene symbols, are shown in **Table S1**. Next, we used a series of filtering steps, as described previously, to identify those genes exhibiting the greatest organ selective expression profile. [21] First, for each organ, probesets expressed greater than 10-fold over the mean of all other tissues were identified. For most tissues, this represented approximately 5–10% of the original number of significant probesets. The extreme cases were pancreas in which only 1% of the probesets achieved this threshold and liver in which 14% of the probesets were greater than 10-fold over the mean of all other tissues. A second filtering threshold, no greater than 3-fold expression over the mean in any other tissue, was defined to arrive at a final list of the most organ defining genes (**Fig. 1C**). Lymph node had the smallest number of selective probesets (24), whereas brain exhibited the greatest number (512). The spleen shares many overlapping cell types and immune functions with lymph node and accordingly, shares a similar gene expression signature (**Fig. 1A and B**). Functionally, the brain (cerebrum in this study) represents the most anatomically and physiologically unique structure, therefore it is not surprising to find the greatest number of tissue selective genes expressed in this tissue. As expected, top organ defining genes included those previously associated with the physiologic function of that organ (Lung example: **Table S2**).

### Validation of Microarray Derived Organ Defining Genes through Quantitative RT-PCR

Quantitative RT-PCR was used to validate the microarray results. Genes were selected for validation, which were described in the previous analysis as organ defining (UMOD, Uromodulin-kidney; LIPC, Hepatic Lipase-liver; RTN1, Reticulon 1-brain). Transcripts exhibited expected tissue selective expression patterns with differential expression even higher by QT-PCR vs. microarray. (**Fig. S2**). Overall, pattern and magnitude of expression across tissues compared to house keeping controls for each validation gene illustrated concordance across platforms.

### Validation of an Informative Database of Normal Canine Tissues: Functional Assessment of Organ Selective Gene Sets

One of the primary limitations predicted and observed during the course of our analysis was the relative lack of functional annotation for canine probesets and corresponding transcripts using resources such as NetAffx™, Ensembl and Entrez Gene. [22], [23], [24] For example, of the 42,860 non-control probesets on the Affymetrix Canine Version 2.0 GeneChip®, only 2,726 (6.4%) have at least one GO term associated. The frequency of GO term annotation is even lower when examining the tissue selective probeset lists such as canine brain where, out of 512 probesets, only 18 (3.5%) are currently annotated with GO terms. This limits the ability to get significant results when using default canine transcript annotations for functional analysis of overrepresented GO terms.

In order to overcome these limitations, we used an alternative approach to more completely annotate canine transcript information. Blast2GO-FAR (B2G-FAR) is a species-centered functional annotation repository enabling whole genome and Affymetrix platform specific transcript annotation. [22], [23], [24], [25] To the best of our knowledge, this resource provides the most extensive high-quality canine probeset and transcript annotations. Compared to NetAffx™ (6.4% probeset annotation), the B2G-FAR annotation file contains GO terms for 49.4% of all non-control probesets and more than 142,000 annotations total for the canine array. Next, we conducted functional enrichment analysis using tissue selective probeset lists for each organ. Analysis was conducted in Blast2GO, which uses GOSSIP to perform a one-sided Fisher's exact test with a modified false discovery rate (FDR) or family wise error rate (FWER) calculation to correct for multiple testing. [26] B2G-FAR annotations were used for the functional analysis of canine tissue selective probeset lists and complete results for the brain are shown in **Table S3**. For each organ, the top overrepresented GO terms (FDR 0.05) described known functions for the tissue as expected. For example, the brain selective probeset list was overrepresented by GO terms such as neurogenesis, synaptic transmission, neuron projection and other neuronal associated functions and processes. Canine kidney was largely characterized by GO terms describing anion/cation transport as well as brush border and other membrane related terms. The immune organs spleen and lymph node were analyzed together and, as expected, were described by GO terms such as immune response, chemokine activity and response to stress. Pancreas was characterized by GO terms representing digestion, cholinesterase and other enzymatic activity as well as extracellular region/space. The most specific liver associated GO terms were microsome/ER membrane, heparin and heme binding and complement activation, classical pathway. Canine jejunum was described by GO terms such as apical plasma membrane, intestinal absorption, microvillar actin bundle formation and various transporter related functions as expected for this organ. Canine lung was characterized by the fewest number of overrepresented GO terms, primarily regulation of liquid surface tension, respiratory gaseous exchange and extracellular region/space. Notably, the heart selective gene list specifically described cardiac functions which did not overlap with the skeletal muscle tissue selective gene list even though both tissues share many other overlapping GO terms representing general striated muscle function and striated muscle components.

Taken together, the results of our gene expression and functional analysis suggest that the canine normal tissue dataset accurately reflects a biologically meaningful transcriptional profile for each tissue. Furthermore, the analysis of the canine data set using B2G-FAR supports the value of this investigative tool for the functional annotation of data sets where complete conventional annotation is not yet available.

### Cross-Species Comparisons Between Canine and Human Orthologs

Orthologous genes are derived from a common ancestral gene and retain similar function. Therefore, it is expected that a comparison of canine and human orthologous gene expression, in matched tissues, should result in clustering based on tissue rather than by species. In order to test this hypothesis, we analyzed Affymetrix human U133A raw data previously published as part of the Novartis Human Normal Tissue Compendium. [20] In cases where there were no matched tissues (jejunum and spleen), raw data in the form of .CEL files were collected from the Gene Expression Omnibus (GEO). [27] Expression values for the human normal tissue data set were determined using identical analysis parameters to the canine normal tissue data (see Methods). Next, both the human and canine data sets were filtered to retain only best sequence matched orthologous probesets, as defined using the Affymetrix Netaffx™ website. In cases where there were more than one probeset representing the same gene symbol, the maximum expression value was used so that there was only a single expression measure for each gene. Expression measures for each gene were z-score transformed for each species independently to allow for subsequent comparison on the same scale. The two datasets were then merged by matching gene symbols resulting in standardized expression measures for 2,598 transcripts.

**Figure 2:**
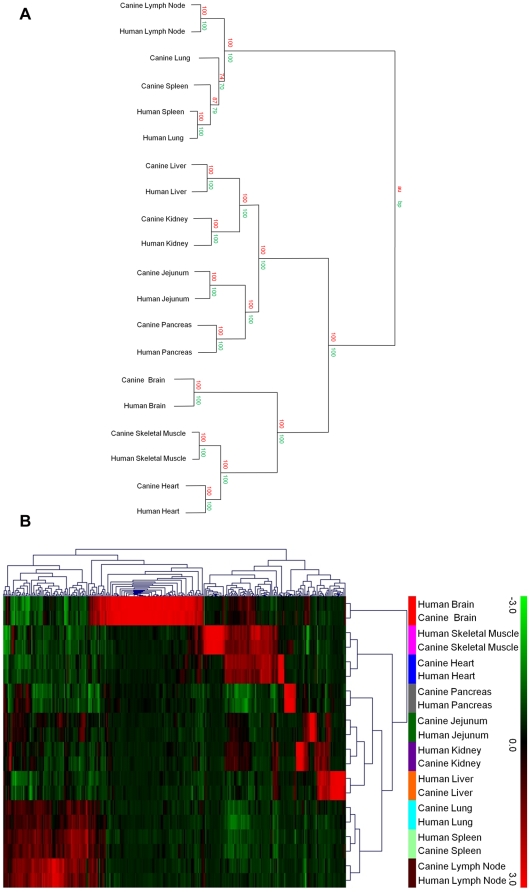
Hierarchical clustering defines relationships between canine and human normal tissues. Orthologous probesets from canine and human Affymetrix gene expression platforms were mapped using NetAffx™ “best sequence” matches. In cases where there were multiple probesets representing the same gene symbol, the one with highest expression was used. This resulted in a total of 2,598 expression measures for comparison between species. No prior information about differential expression was used. The only filtering done was to exclude probesets in each species that were not expressed in at least one tissue. **A**. Hierarchical clustering of canine and human matched tissues based on 2,598 sequence matched orthologous probesets. Sample distances were calculated using Pearson correlation metrics and clusters joined using Ward linkage. Bootstrap re-sampling was conducted (10,000 iterations) in order to determine cluster stability. Confidence measures for multi-level bootstrap analysis are based on approximately unbiased p-values (AU), and simple bootstrap analysis probabilities (BP) for each node of the dendrogram, which are labeled numerically. **B**. Hierarchical clustering of samples and genes was conducted using 294 probesets differentially expressed in at least one tissue based on multi-factor ANOVA (species and tissue). Euclidean distance measure and complete linkage was used for clustering. Within the heatmap, red denotes greater relative expression whereas green denotes lower.

As shown in **Fig. 2A**, hierarchical clustering with bootstrap resampling revealed that samples mainly grouped together based on tissue type rather than by species. In addition, sample grouping was consistent with overlapping anatomical functions and/or cellular composition. For example, lymph node, spleen and lung grouped together in a clade separate from all other tissues (branch point 13). These tissues grouped similarly based on shared expression of genes involved in immune response/functions. Canine lung and spleen were the only two examples of ambiguous cluster assignment at the final branch point. Brain, skeletal muscle and heart also form a distinct clade (branch point 17) while kidney, liver, pancreas and jejunum group together in a final cluster (branch point 16). These results are consistent with our previous hierarchical cluster analysis using all canine tissue replicates and more than 10,000 probesets (**Fig. 1B**). In addition, these results suggest that orthologous canine and human genes share similar tissue enriched and/or tissue selective expression patterns. Next, a multi-factor ANOVA was conducted in order to determine genes differentially expressed based on tissue. After correcting for multiple testing (FDR = 0.001), this resulted in the identification of 294 transcripts which were then analyzed by hierarchical clustering to find tissue enriched and tissue specific orthologous gene clusters. As shown in **Fig. 2B**, the overall structure of sample clustering remained the same with the notable exception being brain, which now separated into a distinct branch due to the high number of very tissue selective transcripts compared to all other tissues. Taken together, this comparative analysis provides further validation of the quality and consistency of the canine expression dataset and suggests the opportunity to add value to the data set from cross-species analysis.

### Using Normal Tissue Gene Expression Data and Comparative Genomics to Redefine Affymetrix Canine Probeset Annotation

**Figure 3:**
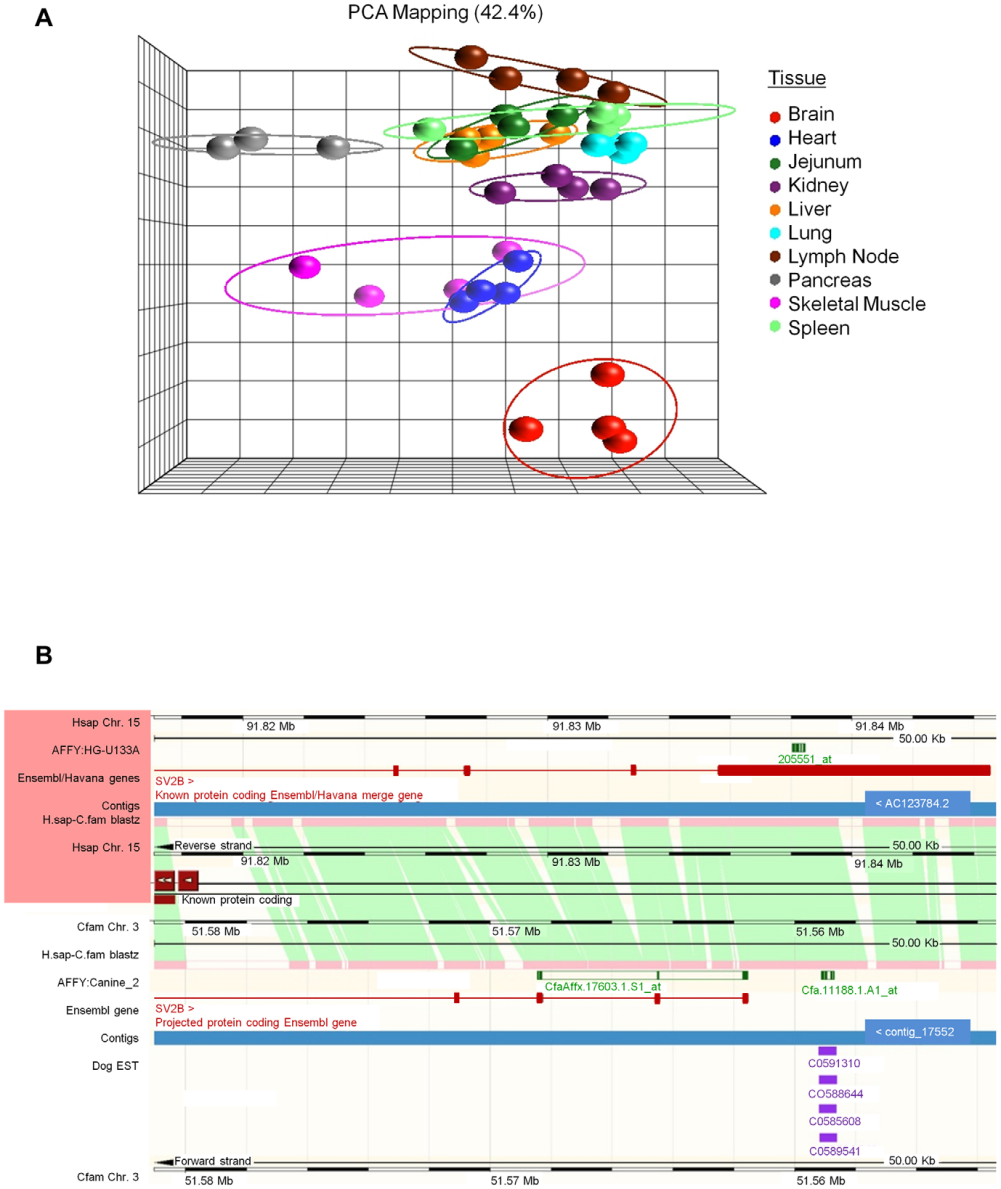
Resolution of transcript assignment for canine probesets mapping to the *SV2B* gene locus. **A**. Principle component analysis of all 11,339 canine probesets with no gene identifier associated. **B**. Ensembl BLASTZ pairwise genomic alignment of human chromosome 15 (top panel) and canine chromosome 3 (bottom panel) centered on the 3′ region of the *SV2B* gene locus. Affymetrix human U133A probeset, 205551_at (SV2B), and canine_2 probesets, CfaAffx.17603.1.S1_at (SV2B) and Cfa.11188.1.A1_at (unidentified) are aligned to their corresponding genomic regions. Canine EST evidence is shown in purple.

A limitation encountered during our study was the large number of probesets for which no canine gene has been assigned (**Table S1**). Out of the 42,860 total probesets on the canine version 2.0 array, 11,339 (27%) have no gene symbol or gene name attributed using NetAffx™ annotations. In order to demonstrate that these unidentified probesets, alone, can provide important information regarding tissue selective gene expression we conducted principle component analysis. As shown in **Fig. 3A**, these probesets, without any prior filtering, were able to clearly separate sample replicates based on tissue. This was especially true for canine brain, pancreas, skeletal muscle and heart. However, it is unclear which transcripts these probesets are assessing and since many are highly expressed in a tissue selective manner it would be of interest to know this information. As a proof of concept, we developed an approach to re-map a subset of the top brain selective probesets for which no gene identifying information was available. This serves to describe a process for further annotation of canine transcripts and genes across all tissues, normal and diseased.

First, Affymetrix probeset identifiers were used to query Ensembl where genomic and transcript linked information was gathered. For example, canine probeset, Cfa.11188.1.A1_at, aligned to a region immediately 3′ to, but not included as part of, the predicted canine *SV2B* gene (**Fig. 3B**). This may explain the lack of gene symbol, gene name or GO term annotations using Netaffx™. Next, a BLASTZ pairwise alignment between the canine and human genomic sequences revealed this region to be syntenic to human chromosome 15. [28] Additional features were mapped to the pairwise alignment including the position of other canine or human probesets for the locus as well as canine EST evidence and known human transcripts.

**Figure 4:**
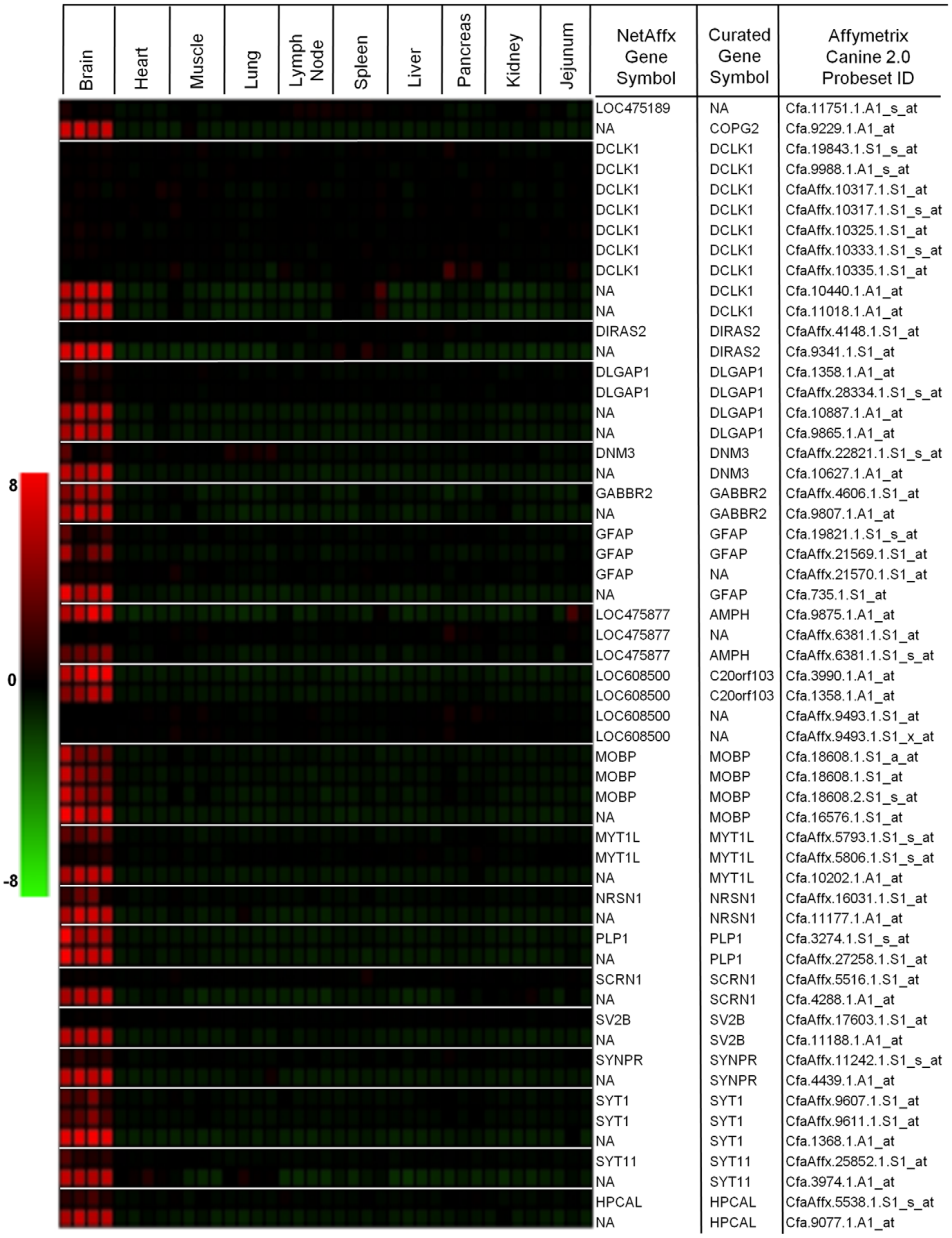
Use of canine-human comparative genomics and expression analysis to improve annotation of canine probesets. Canine brain had the greatest number of probesets without a gene symbol or gene name assigned. As proof-of-concept, these probesets within the top 50 canine brain selective list were re-mapped and underwent manual curation to link transcript information to expression data. Heatmap representing expression values for NetAffx™ unassigned canine probesets (NA) as well as LOC designated probesets in the top 50 brain selective expression list. Following manual curation the newly assigned gene symbols (curated gene symbol) are shown. Once these probesets were mapped to a known transcript, all other canine probesets for that transcript on the canine version 2.0 array were subsequently re-mapped and their relative expression is shown for comparison. In cases where the curated gene symbol is represented by (NA), this denotes that the probeset mapped unambiguously to an intronic region. Red represents increased and green decreased log-fold expression compared to the mean of all other tissues.

*SV2B*, synaptic vesicle glycoprotein 2B, is a known 1-to-1 canine/human ortholog listed in both the Ensembl and Homologene databases and shares 91.9% nucleotide sequence identity and 95.6% at the amino acid level for the predicted protein product [24]. Closer inspection of the gene structure, including intron/exon boundries and non-coding sequences, revealed the primary difference between the two gene annotations was the shorter predicted length of the 3′ untranslated region (UTR) in canine, even though this region is highly conserved. The automated gene annotation process currently employed by Ensembl uses a default UTR length, calculated as the highest of either the mean or the median of all annotated UTRs for a given species [22]. However, multiple lines of EST and ortholog expression evidence suggest the canine *SV2B* 3′ untranslated region may extend further than predicted. Through Bio-GPS/Novartis Symatlas (http://biogps.gnf.org) physical mapping of human *SV2B* exhibits a highly brain specific expression pattern. In addition, canine probeset, Cfa.11188.1.A1_at, physically aligns to the same orthologous region as the human Affymetrix U133A probeset 205551_at. Interestingly, NetAffx™ does list a different canine probeset for the *SV2B* gene, CfaAffx.17603.1.S1_at, which maps to the predicted protein coding sequence. However, only 10/11 probes match the CanFam 2.0 genome assembly and the individual probes are spread out over multiple exons (**Fig. 3B**). In addition, the expression values for this probeset in all canine samples, regardless of tissue, are extremely low (**Fig. 4**). One possibility is that one or more exons are alternatively spliced resulting in decreased sensitivity for this probeset.

Taken with our assessment of its tissue selective expression pattern, our results support an alternative probeset annotation for the *SV2B* gene. This manual annotation process was repeated for the 21 remaining canine probesets with no gene symbol in the brain top 50 tissue selective probeset list (**Table S2**). A heatmap representing mean centered expression values for these probesets, demonstrating their tissue selectivity, is shown in **Fig. 4**. Additional examples of the manual curation process for these probesets are shown in **Fig. S3, S4, S5, S6**. This was done to demonstrate proof-of-concept for our approach and to provide an example for how this canine normal tissue expression data can be used for comparative genomic studies.

### Establishment of a Canine Normal Tissue Database

A publicly available, web accessible database (Canine Normal Tissue Database) was created from this dataset to allow other researchers to query individual gene expression across canine normal tissues. This database is available through the NCI Comparative Oncology Program website ccr.cancer.gov/resources/cop. Raw data (Affymetrix .cel files) can also be downloaded directly from this site for use as controls in other canine genomic analyses. The Canine Normal Tissue Database was developed to allow parallel viewing of gene expression with the (human) Oncogenomics Normal Tissue database, available via a web-based interface described previously ntddb.abcc.ncifcrf.gov/cgi-bin/nltissue.pl [15].

## Discussion

This work represents the first compiled dataset of canine normal organ gene expression profiles. In order to provide a descriptive dataset we used several biological replicates per organ representing both pure bred (beagle) and mixed breed dogs of both sexes and of varying ages. The ten tissues chosen for this study represent a broad selection of organs that are informative across research communities involved in canine health and disease as well as those interested in toxicogenomics or comparative genomics.

The informative value of this canine normal tissue expression dataset was supported in several ways. Organ defining expression data and functional analysis distinguished normal canine tissues. Analysis of expression profiles, by principle component analysis and bootstrapped hierarchical clustering, established that biological replicates grouped together based on tissue of origin demonstrating internal consistency in the dataset. In addition, each organ clustered in a manner consistent with anatomical function and/or cellular composition, similar to what has been published previously for human studies [12], [13], [14], [15]. These results were further supported by our analysis of canine and human orthologous gene expression which demonstrated reproducible clustering based on organ type driven by tissue enriched and tissue selective gene expression profiles. The identification of gene expression profiles with similarity to published human data allowed us to further demonstrate the value of this dataset by informing canine gene and probeset annotation. Exemplified by our annotation of brain specific transcripts, we demonstrated that gene identifiers can now be more confidently linked to previously unknown probesets so as to yield a more complete and functional view of canine gene expression across all tissues.

To enable researchers' access to this comprehensive dataset, a web accessible database was constructed. This allows users to quickly and easily evaluate gene expression across canine tissues using various gene identifiers. In addition, comparative gene expression analysis can be conducted between canine and human normal tissue gene expression. Raw data is also available from this web interface and can be used as uniform normal tissue controls and comparators in future canine genomic analyses as well as for end user specific pre-processing options. Use of the Affymetrix Canine Version 2.0 GeneChip® platform for this study enables a standardized approach for further database growth. Additional samples can easily be incorporated in the future including an expanded repertoire of organ samples as well as experimental data representing diseased tissue from matched canine organs. This gene expression dataset will be of interest to both basic and translational scientists interested in understanding canine health and disease and to advance the dog as a post-genomic species used in biomedical research.

The public release of the canine genome draft in 2005 was pivotal in advancing the study of disease in the dog by broadening the opportunity for advanced high throughput “omic” analyses [2]. This genomic sequencing data, together with the development of the Affymetrix Canine Version 2.0 GeneChip®, provided the opportunity to develop a canine normal tissue gene expression database. One of the goals of the National Cancer Institute's Comparative Oncology Program (NCI-COP) is to include companion animals with cancer in the mainstream of cancer research [1], [5], [29]. One of the mechanisms for accomplishing this goal is through careful genomic and proteomic analysis of cancers in dogs. Interest by academia, industry and regulatory agencies has fueled new collaborations between the human and animal health communities to understand and treat cancer in the dog. These expanding relationships provide an opportunity for a win-win outcome for dogs and humans. We believe the establishment of a canine normal tissue expression database and its public availability will play an integral role in advancing the study of canine disease as well as enable inter-species comparisons of common diseases. In doing so, this promises to improve the health of dogs and humans with cancer and other diseases. [8], [30]

The need for canine normal tissue expression database emerged from challenges presented to us in our own work in comparative genomics, initially in osteosarcoma [31]. The interpretation of comparative genomic data requires an understanding of the gene expression profiles of normal human and canine tissues. Furthermore, improvements in gene and transcript annotation are necessary to more completely define data emerging from gene expression experiments. The opportunity to rapidly query an existing and robustly annotated database of normal canine tissues would have hastened the completion of our studies and substantially reduced costs. Our previous work is an example of many similar studies that would benefit from a common characterization of canine normal tissues. [7], [9], [32], [33], [34]

In summary, this work establishes a validated database of canine normal tissue gene expression data making genomic characterization of diseased states in the dog, like cancer and others, less expensive and expedient. This publicly available database can be queried for canine and human gene expression patterns across matched normal tissues. It is our expectation that this data will lead to improvements in our understanding of diseases and conditions that afflict both dogs and humans.

## Materials and Methods

### Canine tissue samples

Forty (40) pathologically normal organ samples were collected from four dogs, 10 organs from each dog. They included two males and two females ranging in age from 3 months to 6 years of age. Two of the animals were beagles and two were mixed breed dogs. Organ samples included the pancreas, kidney, liver, lung, heart, skeletal muscle, jejunum, cerebrum, spleen and peripheral lymph node. Samples were collected and frozen in RNAlater® within 30 minutes of collection. All samples were stored at – 80°C until processing.

### RNA extraction and Affymetrix microarray processing

RNA was extracted from all samples using TRIzol® according to manufacturers instructions (Invitrogen, Carlsbad, CA). Samples were then further purified using Qiagen miniprep RNA clean up (Qiagen, Valencia, CA) as per Affymetrix protocols for array preparation. Samples were quantified and assessed for quality using a Bioanalyzer 2100 instrument (Agilent Technologies, Palo Alto, CA). Five micrograms of purified RNA was reverse transcribed and used to make cRNA. Samples were hybridized to Canine Genome Version 2.0 Affymetrix oligonucleotide arrays according to manufacturers instructions (Affymetrix, Santa Clara, CA) at the NCI Microarray Core Facility (Frederick, MD).

### Analysis of canine differential gene expression

Affymetrix .cel files containing the raw, probe level signal intensities, were analyzed using Partek® software, version 6.4 (build 6.09.0310, Copyright 1993–2009, Partek Inc. Partek and all other Partek Inc. product or service names are registered trademarks or trademarks of Partek Inc., St. Louis, MO, USA.). Robust multichip averaging (RMA) was used for pre-processing of probe level data (using only interrogating probes) including pre-background adjustment for GC content and probe sequence followed by RMA background correction. All chips underwent quantile normalization and probeset summaries were median polished [35].

Analysis of variance (ANOVA) was used to determine statistically significant differences in probeset signal intensities between organ types. Categorical variables included in the ANOVA model were tissue type and scan date (random variable). Contrasts included single tissues versus the remaining nine tissues. Adjusted p-values were obtained using a Benjamini-Hochberg step-up procedure to control the false discovery rate (FDR) (set at 0.001) [36]. Least-square mean signal intensities were used in the analysis in order to account for the unbalanced experimental design following exclusion of the single pancreas sample. Lists of differentially expressed probesets for each organ were generated based on FDR corrected p-values obtained from contrasts built into the ANOVA model. These lists were further filtered to identify tissue selective genes using an arbitrary cut-off of greater than 10-fold increased in one organ compared to the median expression value in all other organs and no greater than 3-fold over mean expression in any other tissue.

### Principle component analysis

To analyze the relationships of all canine sample replicates as a means of identifying outliers, we conducted principle component analysis on all samples and probesets initially (**Fig. S1**). This assessment revealed a single sample (one pancreas sample) to be an outlier based on the relative distribution of all other replicates for each tissue as well as the other three biological replicates for the pancreas and lack of expression of classic pancreatic genes such as insulin, pancreatic secretory trypsin inhibitor precursor, elastase 1 and carboxypeptidase B1 (data not shown). Therefore, we repeated the Affymetrix .cel file import process using the remaining 39 samples and re-processed the data as above.

### Quantitative RT-PCR Validation

For quantitative RT-PCR validation of microarray results, an independent set of RNA from each of nine tissues was purchased commercially (Zyagen Laboratories, San Diego, CA). cDNA synthesis reactions were conducted using 100ng of total RNA and reverse transcribed using the iScriptTM cDNA synthesis kit according to manufacturers instructions (Bio-Rad, Hercules, CA). Diluted cDNA (1∶3) was used as template for Taqman based PCR reactions using the iQTM Supermix according to manufacurers instructions (Bio-Rad, Hercules, CA). The final concentration of each primer was 500 nM and each probe 250 nM for all primer/probe combinations. PCR reactions were conducted using an iQ5 thermocycler (Bio-Rad, Hercules, CA). Thermocycling conditions were as follows: 95C for 3 minutes followed by 35 cycles of 95C for 5 seconds and 60C for 25 seconds. For quantitation, samples were normalized using 18S as the reference and each sample was compared to the group mean threshold cycle value in order to determine normalized, log-fold expression. [37] The following transcript specific oligonucleotide primers and 5′-6-FAM-internal ZEN-3′-Iowa Black® probes were synthesized by Integrated DNA Technologies (Coralville, Iowa): Gene: Uromodulin (UMOD)

Probe: 5′-/56-FAM/ATCATAGAC/ZEN/CAAAGCCGCGTCCTG/3IABKFQ/-3′

Primer1: 5′-TGTGAAGTGTATCTCTGCGAC-3′

Primer2: 5′-CCTTGAGACCACTGCCTG-3′

Gene: Hepatic Lipase (LIPC)

Probe: 5′-/56-FAM/TCCCCCAAA/ZEN/CCCAGGAGAAAACC/3IABKFQ/-3′

Primer1: 5′-AGAGTTTATGTCGCACCTCAC-3′

Primer2: 5′-CACCATCAAAGTCAAAGCAGG-3′

Gene: Reticulon 1 (RTN1)

Probe: 5′-/56-FAM/CCTTTTAGC/ZEN/GCCTGGGATTTTAGCCT/3IABKFQ/-3′

Primer1: 5′-ACCCGTAGTGTATGTTAAGCAC-3′

Primer2: 5′-GGTGGGACATCGATTTACTCAG-3′

Gene: 18S

Probe: 5′-/56-FAM/GCGACGACC/ZEN/CATTCGAACGT/3IABKFQ/-3′

Primer1: 5′-TTTGGTGACTCTAGATAACCTCGGGC-3′

Primer2: 5′-ACCATCGAAAGTTGATAGGGCAG-3′

### Hierarchical clustering

Agglomerative hierarchical clustering of biological replicates was done following ANOVA using probesets filtered on FDR (0.001) corrected p-values. In cases where there were multiple probesets for the same gene symbol, the maximum intensity value was used to arrive at a single expression measure for each gene. Gene expression values were median centered and normalized to a standard deviation of 1. Between sample and between gene distances were calculated using Pearson dissimilarity as the measure and Ward linkage was used to join clusters unless otherwise noted. For between sample comparisons, bootstrap re-sampling (either 1,000 or 10,000 iterations as indicated) was conducted in order to determine the stability of clustering results using the pvclust package implemented in the R statistical programming environment [38]. Two types of p-values were included in the results. (AU) approximately unbiased values were derived from multi-scale bootstrap analysis and (BP) bootstrap probability values were derived from normal bootstrap re-sampling.

### Genome database information used for this study

For analysis in Ensembl, UCSC and NCBI, information from the human genome build GRCh37 (hg18) was used. For canine, the CanFam2.0 canine genome assembly was used. Canine-human genome alignments were generated by pairwise BLASTZ alignment and visualized using Ensembl tools [22], [28]. Syntenic regions of canine and human chromosomes were determined using Ensembl as well as through the Broad Institute Alpheus website (http://www.broadinstitute.org/~mclamp/alpheus/).

### Canine gene annotations

Initial canine probeset annotations, including gene ontology (GO) terms and mapping of gene identifiers, were done using the Canine Version 2.0 annotation file (Canine_2.na29.annot.cvs, released July, 2009) from the Affymetrix Netaffx™ website at www.affymetrix.com/netaffx [23]. When indicated in the text, custom annotations for the Affymetrix Canine Version 2.0 GeneChip® were obtained from B2G-FAR, the Blast2GO functional annotation repository at http://bioinfo.cipf.es/b2gfar/affychips:canine [25], [39]. The top 50 canine brain selective probesets without previously assigned gene symbols, or those having ambiguous gene descriptors were mapped using ENSMBL probeset to genome alignments followed by manual curation. Individual probesets were analyzed for: (1) unambiguous alignment to the canine genome; (2) mapping to syntenic regions based on canine-human alignment; (3) physical mapping to matching human Affymetrix probesets where tissue selective expression could be verified through Symatlas at http://biogps.gnf.org; (4) EST based evidence; (5) expression comparison with other previously identified and Affymetrix annotated canine probesets for a given gene locus; (6) polyadenylation signal sequence and 3′UTR alignment with human.

### Functional assessment of tissue selective gene sets

Canine tissue selective probesets, as well as all non-control probesets on the array, were also annotated using B2G-FAR Affymetrix Canine Version 2.0 GeneChip custom annotations [25], [39]. The annotation file was implemented in Blast2GO followed by enrichment analysis using GOSSIP (http://gossip.gene-groups.net) [26]. A one-sided Fisher's exact test was conducted to find over-represented GO terms for the canine brain (test list) using the rest of the array as the reference list. Results are reported with uncorrected p-values as well as values adjusted for multiple comparison using false discovery rate and family wise error rate as described at http://home.clara.net/sisa/fishrhlp.htm.

### Comparison of canine and human orthologous gene expression

In order to analyze data between different Affymetrix platforms and species, canine-to-human ortholog probeset matches were identified using the Affymetrix Netaffx™ website at www.affymetrix.com/netaffx. Probesets from the Affymetrix Canine Version 2.0 GeneChip® were mapped to human orthologous probesets on the Affymetrix Human HG-U133A GeneChip® to allow for cross-species comparisons of matched organs using publicly available human gene expression data obtained via the Gene Expression Omnibus (GEO) at http://www.ncbi.nlm.nih.gov/geo [4]. Human brain GSM44690, human liver GSM35982, human spleen GSM35999, human skeletal muscle GSM244532, human heart GSM44671, human jejunum GSM44679, human kidney GSM44675, human lung GSM35985, human liver GSM51371, human pancreas GSM18977. Human .cel file data for each organ was pre-processed as described for canine data using using Partek® software, version 6.4. The final list of best sequence matched, orthologous probesets, as defined by Affymetrix NetAffx™, for canine and human were consolidated based on matching gene symbols using maximum expression values in cases where more than one probeset matched the same gene symbol. In addition, for each species, each probeset was considered for analysis only if there was demonstration of expression in at least one tissue. This resulted in a final list of 2,598 expression measures for comparison. The signal intensities for each species were then standardized independently using z-score transformation and then the data from both species was merged to a single dataset.

## Supporting Information

Figure S1 **Principle component analysis define relationships between canine normal tissues and identifies one pancreas outlier.** mRNA expression for 40 samples from ten pathologically normal canine tissues were analyzed using the Affymetrix Canine Version 2.0 GeneChip®. Only probesets differentially expressed in at least one tissue (as described in the Methods) were included in the analysis. Each sphere represents an individual sample, colored by tissue and ellipses correspond to two standard deviations of the tissue group mean. A single pancreas sample was excluded from further analysis.
(TIF)

Figure S2 **Log-fold difference compared to the group (all tissues) mean for three organ-defining genes via quantitative RT-PCR validates microarray data results.** Genes selected for microarray results validation were previously described as organ defining (UMOD, Uromodulin-kidney; LIPC, Hepatic Lipase-liver; RTN1, Reticulon 1-brain). UMOD expression in the canine kidney is 13.6 fold higher, LIPC expression 8.0 fold higher in the canine liver, and RTN1 expression 11.1 fold higher in the canine cerbreal cortex via QT-PCR than the group mean of all other canine tissues. Transcripts exhibited expected tissue selective expression patterns with differential expression even higher by QT-PCR vs. microarray.
(TIF)

Figure S3 **Resolution of transcript assignment for canine probesets mapping to the AMPH gene locus.** **A**. Ensembl synteny map of canine chromosome 18 and human chromosome 7 highlighting the SV2B gene locus in each species. **B**. Ensembl BLASTZ genomic alignment of human chromosome 7 (top panel) and canine chromosome 18 (bottom panel) centered on the 3′ region of the amphiphysin (AMPH) gene locus. Affymetrix human U133A probeset, 205257_s_at (AMPH), and canine_2 probesets, Cfa.9875.1.A1_at (unidentified) and CfaAffx.6381.1.S1_s_at (AMPH) are aligned to their corresponding genomic regions. Canine EST evidence is shown in purple.
(TIF)

Figure S4 **Resolution of transcript assignment for canine probesets mapping to the DNM3 gene locus.** **A**. Ensembl synteny map of canine chromosome 7 and human chromosome 1 highlighting the dynamin 3 (DNM3) gene locus in each species. **B**. Ensembl BLASTZ genomic alignment of human chromosome 1 (top panel) and canine chromosome 7 (bottom panel) centered on the 3′ region of the DNM3 gene locus. Affymetrix human U133A probeset, 209839_at (DNM3), and canine_2 probeset, Cfa.10627.1.A1_at (unidentified) are aligned to their corresponding genomic regions. Canine EST evidence is shown in purple.
(TIF)

Figure S5 **Resolution of transcript assignment for canine probesets mapping to the DCLK1 gene locus.** **A**. Ensembl synteny map of canine chromosome 25 and human chromosome 13 highlighting the doublecortin-like kinase 1 (DCLK1) gene locus in each species. **B**. Ensembl BLASTZ genomic alignment of human chromosome 13 (top panel) and canine chromosome 25 (bottom panel) centered on the 3′ region of the DCLK1 gene locus. Affymetrix human U133A probesets, 215303_at (DCLK1) and 205399_at (DCLK1), and canine_2 probesets, Cfa.11018.1.A1_at (unidentified), Cfa.10440.1.A1_at (unidentified), CfaAffx.10325.1.S1_at (unidentified), CfaAffx.10346.1.S1_at (DCLK1) and CfaAffx.10333.1.S1_s_at (DCLK1) are aligned to their corresponding genomic regions. Canine EST evidence is shown in purple.
(TIF)

Figure S6 **Resolution of transcript assignment for canine probesets mapping to the NRSN1 gene locus.** **A**. Ensembl synteny map of canine chromosome 35 and human chromosome 6 highlighting the neurensin 1 (NRSN1) gene locus in each species. **B**. Ensembl BLASTZ genomic alignment of human chromosome 6 (top panel) and canine chromosome 35 (bottom panel) centered on the 3′ region of the NRSN1 gene locus. Affymetrix human GNFh probeset, 239293_at (NRSN1) and canine_2 probesets, CfaAffx.16031.1.S1_at (NRSN1), Cfa.11177.1.A1_at (unidentified), are aligned to their corresponding genomic regions. Canine EST evidence is shown in purple.
(TIF)

Table S1 **Differentially expressed probesets and corresponding unique gene symbols in canine tissues.** Describes the number of differentially expressed probesets and corresponding unique gene symbols for each of the ten canine organs examined in this dataset.
(DOC)

Table S2 **Canine lung selective probesets rank ordered with Fold Change vs. all tissues included.** Defines probesets specific to the canine lung, and is an example of tissue selective ranking of probesets conducted for each of the ten canine organs examined in this dataset.
(DOC)

Table S3 **Canine Brain Selective Probesets Over Represented GO Terms.** Defines over-represented GO terms in the canine brain, and is an example of the process used to define GO terms for each of the ten canine organs examined in this dataset.
(DOC)

## References

[1] Khanna C, Lindblad-Toh K, Vail D, London C, Bergman P (2006). The dog as a cancer model.. Nat Biotechnol.

[2] Lindblad-Toh K, Wade CM, Mikkelsen TS, Karlsson EK, Jaffe DB (2005). Genome sequence, comparative analysis and haplotype structure of
the domestic dog.. Nature.

[3] Parker HG, Ostrander EA (2005). Canine genomics and genetics: running with the
pack.. PLoS Genet.

[4] Holzwarth JA, Middleton RP, Roberts M, Mansourian R, Raymond F (2005). The development of a high-density canine
microarray.. J Hered.

[5] Paoloni M, Khanna C (2008). Translation of new cancer treatments from pet dogs to
humans.. Nat Rev Cancer.

[6] Thomas R, Scott A, Langford CF, Fosmire SP, Jubala CM (2005). Construction of a 2-Mb resolution BAC microarray for CGH analysis
of canine tumors.. Genome Res.

[7] Klopfleisch R, Lenze D, Hummel M, Gruber AD (2010). Metastatic canine mammary carcinomas can be identified by a gene
expression profile that partly overlaps with human breast cancer
profiles.. BMC Cancer.

[8] Gallardo-Arrieta F, Doll A, Rigau M, Mogas T, Juanpere N (2010). A transcriptional signature associated with the onset of benign
prostate hyperplasia in a canine model.. Prostate.

[9] Zheng J, Chen Y, Pat B, Dell'italia LA, Tillson M (2009). Microarray identifies extensive downregulation of noncollagen
extracellular matrix and profibrotic growth factor genes in chronic isolated
mitral regurgitation in the dog.. Circulation.

[10] Axelsen JB, Lotem J, Sachs L, Domany E (2007). Genes overexpressed in different human solid cancers exhibit
different tissue-specific expression profiles.. Proc Natl Acad Sci U S A.

[11] Khan J (2003). Genomic & proteomic technological advances in cancer
research.. Pharmacogenomics.

[12] Hsiao LL, Dangond F, Yoshida T, Hong R, Jensen RV (2001). A compendium of gene expression in normal human
tissues.. Physiol Genomics.

[13] Saito-Hisaminato A, Katagiri T, Kakiuchi S, Nakamura T, Tsunoda T (2002). Genome-wide profiling of gene expression in 29 normal human
tissues with a cDNA microarray.. DNA Res.

[14] Shyamsundar R, Kim YH, Higgins JP, Montgomery K, Jorden M (2005). A DNA microarray survey of gene expression in normal human
tissues.. Genome Biol.

[15] Son CG, Bilke S, Davis S, Greer BT, Wei JS (2005). Database of mRNA gene expression profiles of multiple human
organs.. Genome Res.

[16] Hornshoj H, Conley LN, Hedegaard J, Sorensen P, Panitz F (2007). Microarray expression profiles of 20.000 genes across 23 healthy
porcine tissues.. PLoS One.

[17] Kilpinen S, Autio R, Ojala K, Iljin K, Bucher E (2008). Systematic bioinformatic analysis of expression levels of 17,330
human genes across 9,783 samples from 175 types of healthy and pathological
tissues.. Genome Biol.

[18] Shmueli O, Horn-Saban S, Chalifa-Caspi V, Shmoish M, Ophir R (2003). GeneNote: whole genome expression profiles in normal human
tissues.. C R Biol.

[19] Su AI, Cooke MP, Ching KA, Hakak Y, Walker JR (2002). Large-scale analysis of the human and mouse
transcriptomes.. Proc Natl Acad Sci U S A.

[20] Su AI, Wiltshire T, Batalov S, Lapp H, Ching KA (2004). A gene atlas of the mouse and human protein-encoding
transcriptomes.. Proc Natl Acad Sci U S A.

[21] Walker JR, Su AI, Self DW, Hogenesch JB, Lapp H (2004). Applications of a rat multiple tissue gene expression data
set.. Genome Res.

[22] Fernandez-Suarez XM, Schuster MK (2007). Using the Ensembl genome server to browse genomic sequence
data.. Curr Protoc Bioinformatics Chapter 1: Unit 1.

[23] Liu G, Loraine AE, Shigeta R, Cline M, Cheng J (2003). NetAffx: Affymetrix probesets and annotations.. Nucleic Acids Res.

[24] Maglott D, Ostell J, Pruitt KD, Tatusova T (2007). Entrez Gene: gene-centered information at NCBI.. Nucleic Acids Res.

[25] Conesa A, Gotz S, Garcia-Gomez JM, Terol J, Talon M (2005). Blast2GO: a universal tool for annotation, visualization and
analysis in functional genomics research.. Bioinformatics.

[26] Bluthgen N, Brand K, Cajavec B, Swat M, Herzel H (2005). Biological profiling of gene groups utilizing Gene
Ontology.. Genome Inform.

[27] Barrett T, Edgar R (2006). Gene expression omnibus: microarray data storage, submission,
retrieval, and analysis.. Methods Enzymol.

[28] Schwartz S, Kent WJ, Smit A, Zhang Z, Baertsch R (2003). Human-mouse alignments with BLASTZ.. Genome Res.

[29] Paoloni MC, Khanna C (2007). Comparative oncology today.. Vet Clin North Am Small Anim Pract.

[30] Genini S, Zangerl B, Slavik J, Acland GM, Beltran WA (2010). Transcriptional profile analysis of RPGRORF15 frameshift mutation
identifies novel genes associated with retinal degeneration.. Invest Ophthalmol Vis Sci.

[31] Paoloni M, Davis S, Lana S, Withrow S, Sangiorgi L (2009). Canine tumor cross-species genomics uncovers targets linked to
osteosarcoma progression.. BMC Genomics.

[32] O'Donoghue LE, Ptitsyn AA, Kamstock DA, Siebert J, Thomas RS (2010). Expression profiling in canine osteosarcoma: identification of
biomarkers and pathways associated with outcome.. BMC Cancer.

[33] Higgins RJ, Dickinson PJ, LeCouteur RA, Bollen AW, Wang H (2010). Spontaneous canine gliomas: overexpression of EGFR, PDGFRalpha
and IGFBP2 demonstrated by tissue microarray
immunophenotyping.. J Neurooncol.

[34] Sakai D, Nakai T, Mochida J, Alini M, Grad S (2009). Differential phenotype of intervertebral disc cells: microarray
and immunohistochemical analysis of canine nucleus pulposus and anulus
fibrosus.. Spine (Phila Pa 1976).

[35] Bolstad BM, Irizarry RA, Astrand M, Speed TP (2003). A comparison of normalization methods for high density
oligonucleotide array data based on variance and bias.. Bioinformatics.

[36] Hochberg Y, Benjamini Y (1990). More powerful procedures for multiple significance
testing.. Stat Med.

[37] Pfaffl MW (2001). A new mathematical model for relative quantification in real-time
RT-PCR.. Nucleic Acids Res.

[38] Suzuki R, Shimodaira H (2006). Pvclust: an R package for assessing the uncertainty in
hierarchical clustering.. Bioinformatics.

[39] Gotz S, Garcia-Gomez JM, Terol J, Williams TD, Nagaraj SH (2008). High-throughput functional annotation and data mining with the
Blast2GO suite.. Nucleic Acids Res.

