# Supplementary figures and images for: A Compendium of Canine Normal Tissue Gene Expression
Source: PLoS One. 2011 May 31;6(5):e17107. doi: 10.1371/journal.pone.0017107 (PMC3104984; doi:10.1371/journal.pone.0017107)

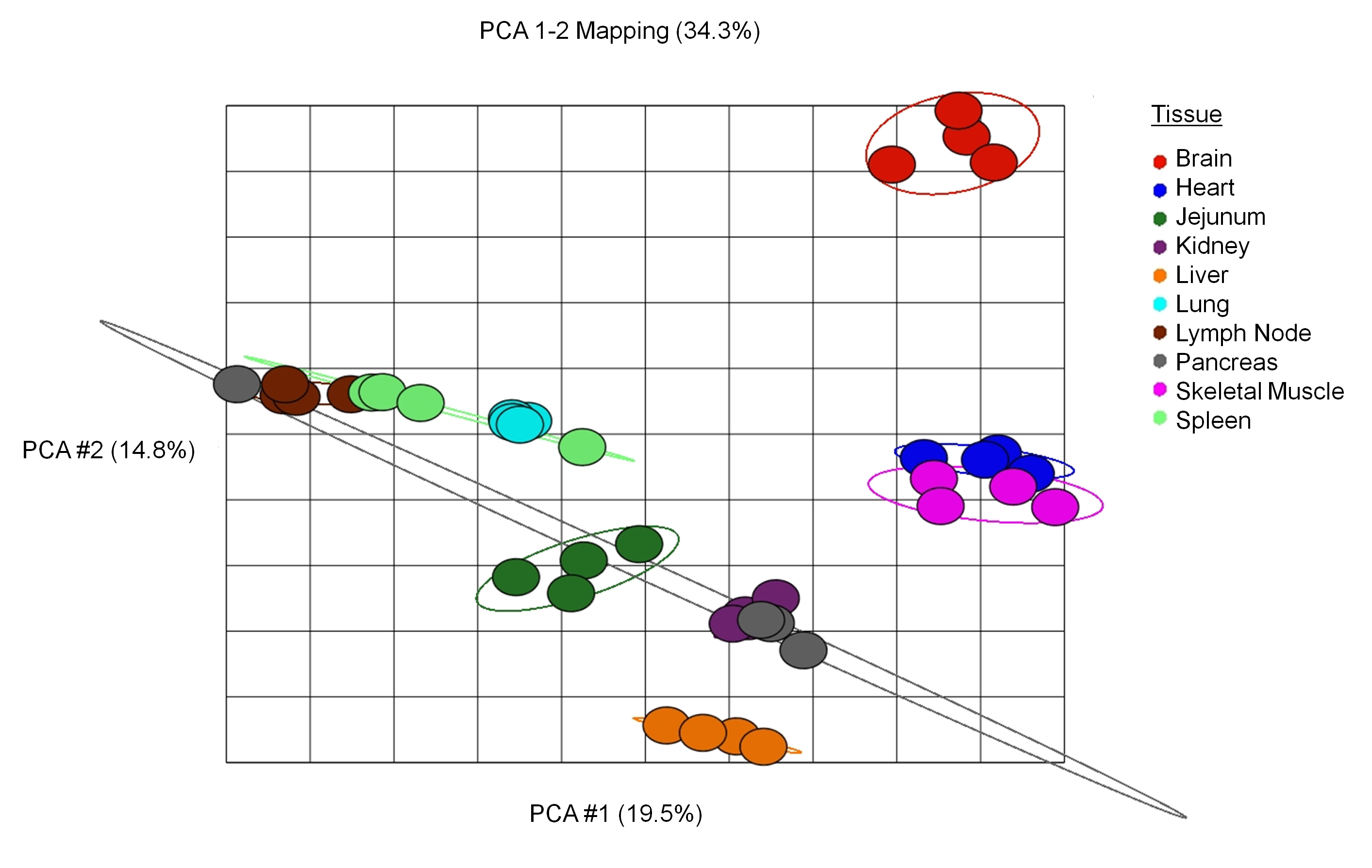

Supplement: Figure S1 — Principle component analysis define relationships between canine normal tissues and identifies one pancreas outlier. mRNA expression for 40 samples from ten pathologically normal canine tissues were analyzed using the Affymetrix Canine Version 2.0 GeneChip®. Only probesets differentially expressed in at least one tissue (as described in the Methods) were included in the analysis. Each sphere represents an individual sample, colored by tissue and ellipses correspond to two standard deviations of the tissue group mean. A single pancreas sample was excluded from further analysis. (TIF) [file pone.0017107.s001.tif]

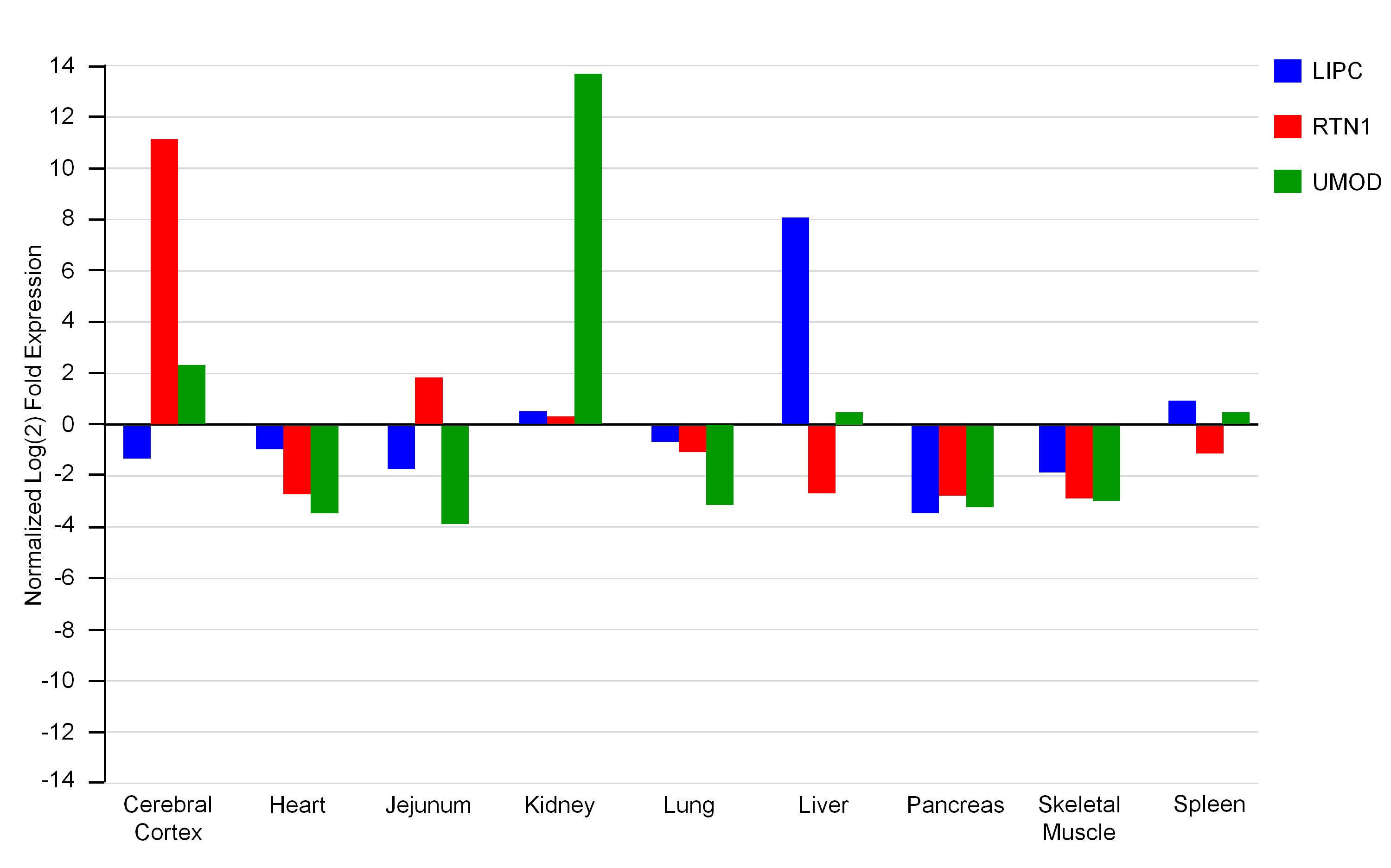

Supplement: Figure S2 — Log-fold difference compared to the group (all tissues) mean for three organ-defining genes via quantitative RT-PCR validates microarray data results. Genes selected for microarray results validation were previously described as organ defining (UMOD, Uromodulin-kidney; LIPC, Hepatic Lipase-liver; RTN1, Reticulon 1-brain). UMOD expression in the canine kidney is 13.6 fold higher, LIPC expression 8.0 fold higher in the canine liver, and RTN1 expression 11.1 fold higher in the canine cerbreal cortex via QT-PCR than the group mean of all other canine tissues. Transcripts exhibited expected tissue selective expression patterns with differential expression even higher by QT-PCR vs. microarray. (TIF) [file pone.0017107.s002.tif]

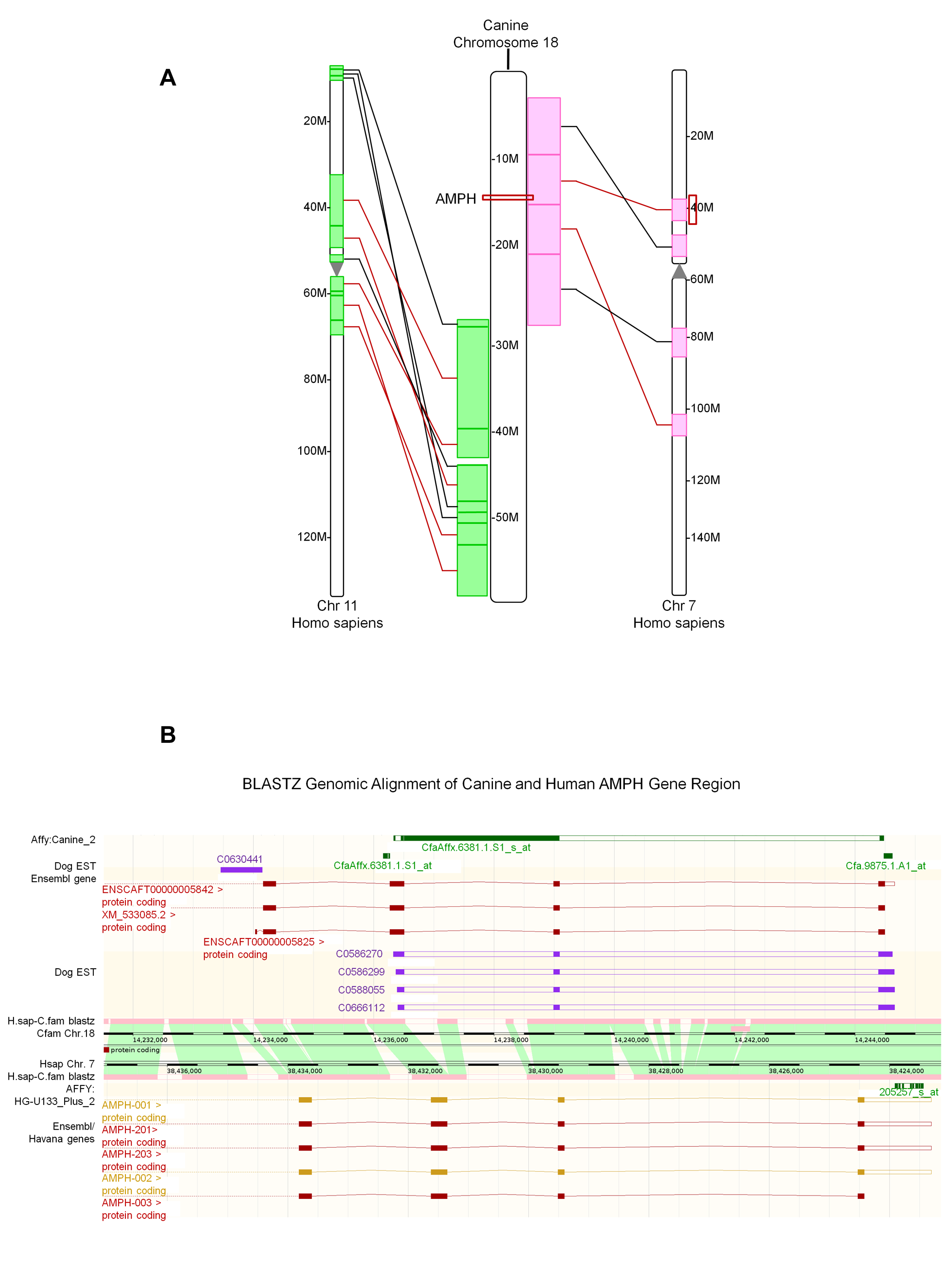

Supplement: Figure S3 — Resolution of transcript assignment for canine probesets mapping to the AMPH gene locus. A. Ensembl synteny map of canine chromosome 18 and human chromosome 7 highlighting the SV2B gene locus in each species. B. Ensembl BLASTZ genomic alignment of human chromosome 7 (top panel) and canine chromosome 18 (bottom panel) centered on the 3′ region of the amphiphysin (AMPH) gene locus. Affymetrix human U133A probeset, 205257_s_at (AMPH), and canine_2 probesets, Cfa.9875.1.A1_at (unidentified) and CfaAffx.6381.1.S1_s_at (AMPH) are aligned to their corresponding genomic regions. Canine EST evidence is shown in purple. (TIF) [file pone.0017107.s003.tif]

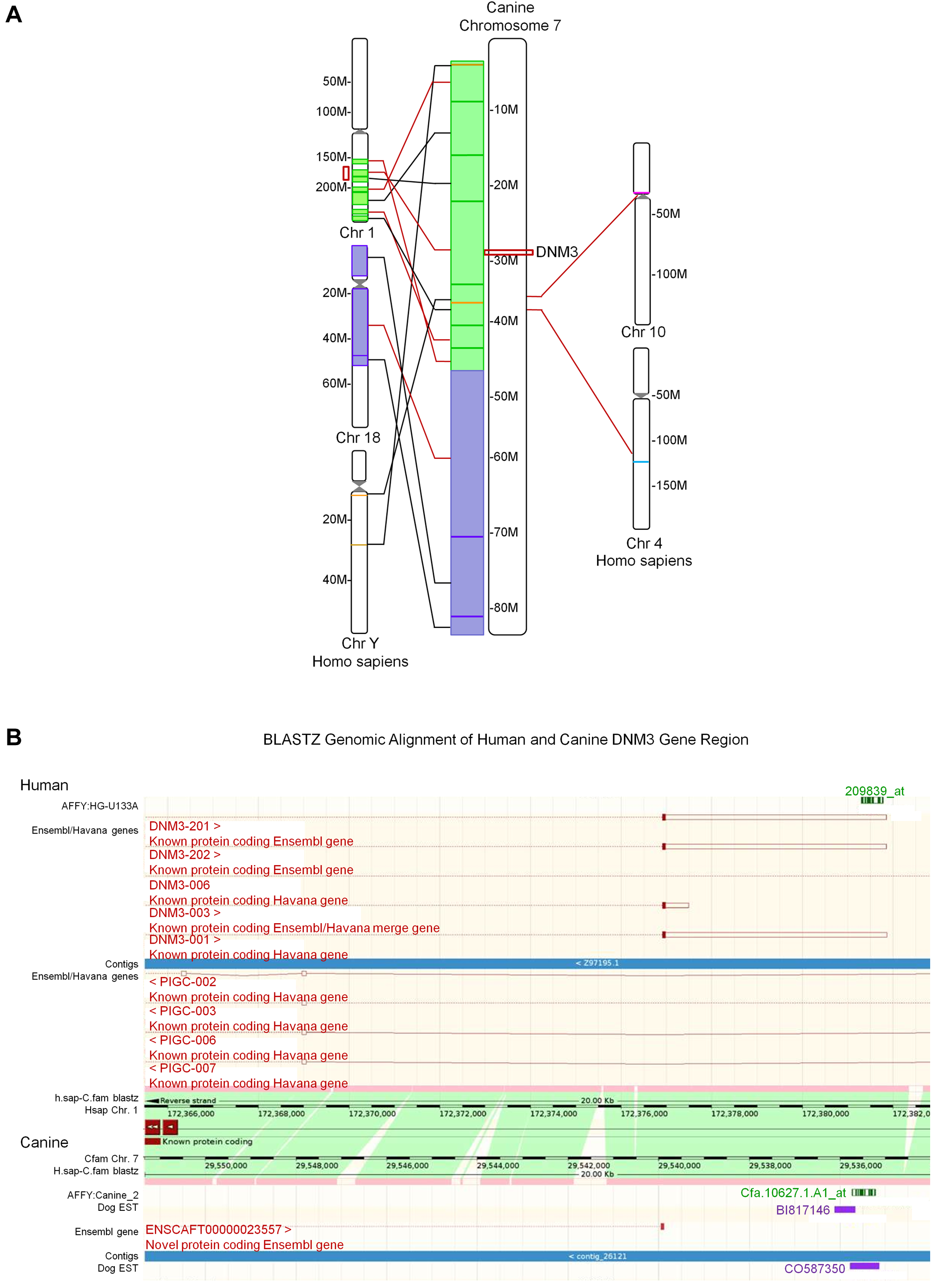

Supplement: Figure S4 — Resolution of transcript assignment for canine probesets mapping to the DNM3 gene locus. A. Ensembl synteny map of canine chromosome 7 and human chromosome 1 highlighting the dynamin 3 (DNM3) gene locus in each species. B. Ensembl BLASTZ genomic alignment of human chromosome 1 (top panel) and canine chromosome 7 (bottom panel) centered on the 3′ region of the DNM3 gene locus. Affymetrix human U133A probeset, 209839_at (DNM3), and canine_2 probeset, Cfa.10627.1.A1_at (unidentified) are aligned to their corresponding genomic regions. Canine EST evidence is shown in purple. (TIF) [file pone.0017107.s004.tif]

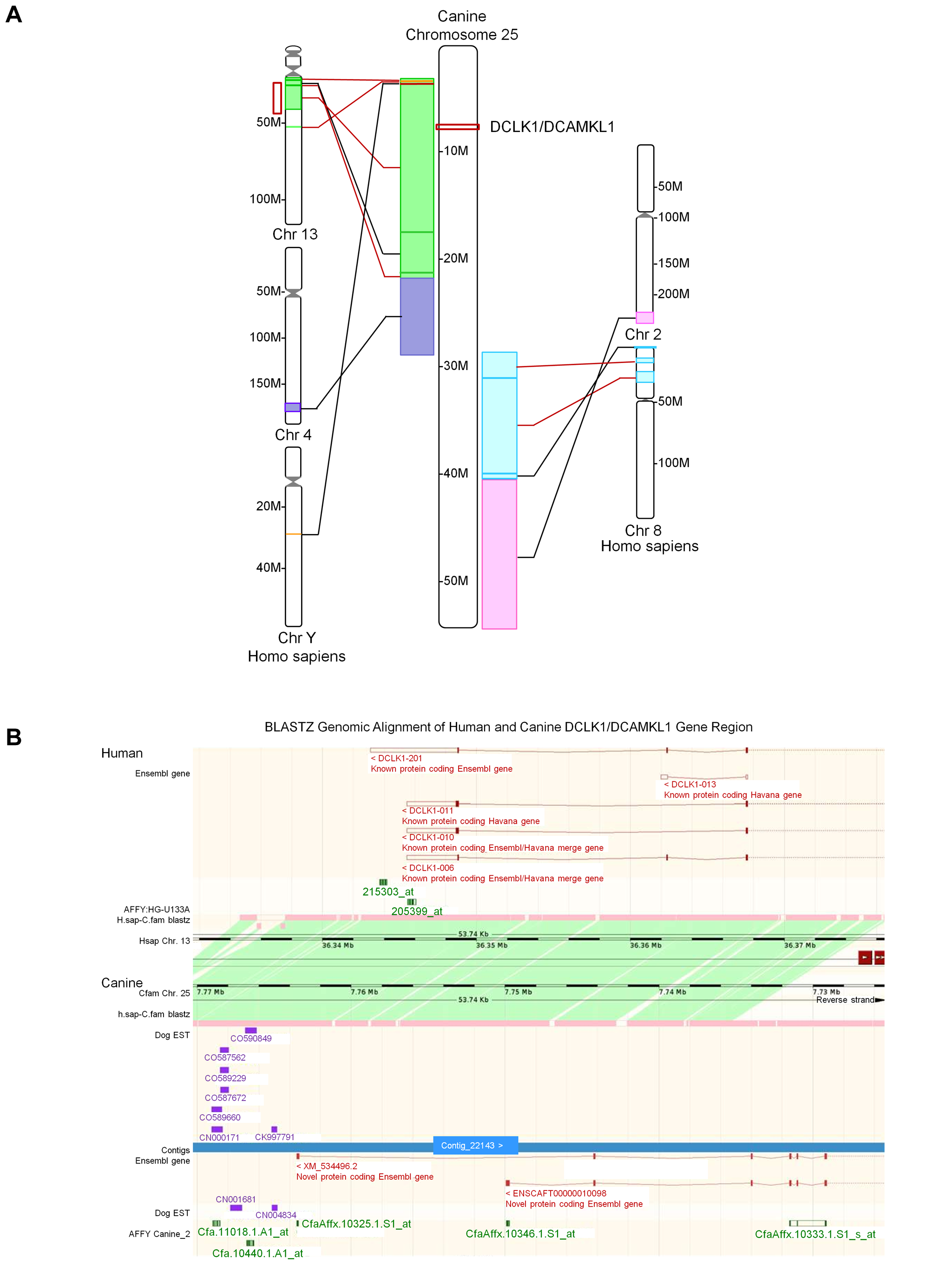

Supplement: Figure S5 — Resolution of transcript assignment for canine probesets mapping to the DCLK1 gene locus. A. Ensembl synteny map of canine chromosome 25 and human chromosome 13 highlighting the doublecortin-like kinase 1 (DCLK1) gene locus in each species. B. Ensembl BLASTZ genomic alignment of human chromosome 13 (top panel) and canine chromosome 25 (bottom panel) centered on the 3′ region of the DCLK1 gene locus. Affymetrix human U133A probesets, 215303_at (DCLK1) and 205399_at (DCLK1), and canine_2 probesets, Cfa.11018.1.A1_at (unidentified), Cfa.10440.1.A1_at (unidentified), CfaAffx.10325.1.S1_at (unidentified), CfaAffx.10346.1.S1_at (DCLK1) and CfaAffx.10333.1.S1_s_at (DCLK1) are aligned to their corresponding genomic regions. Canine EST evidence is shown in purple. (TIF) [file pone.0017107.s005.tif]

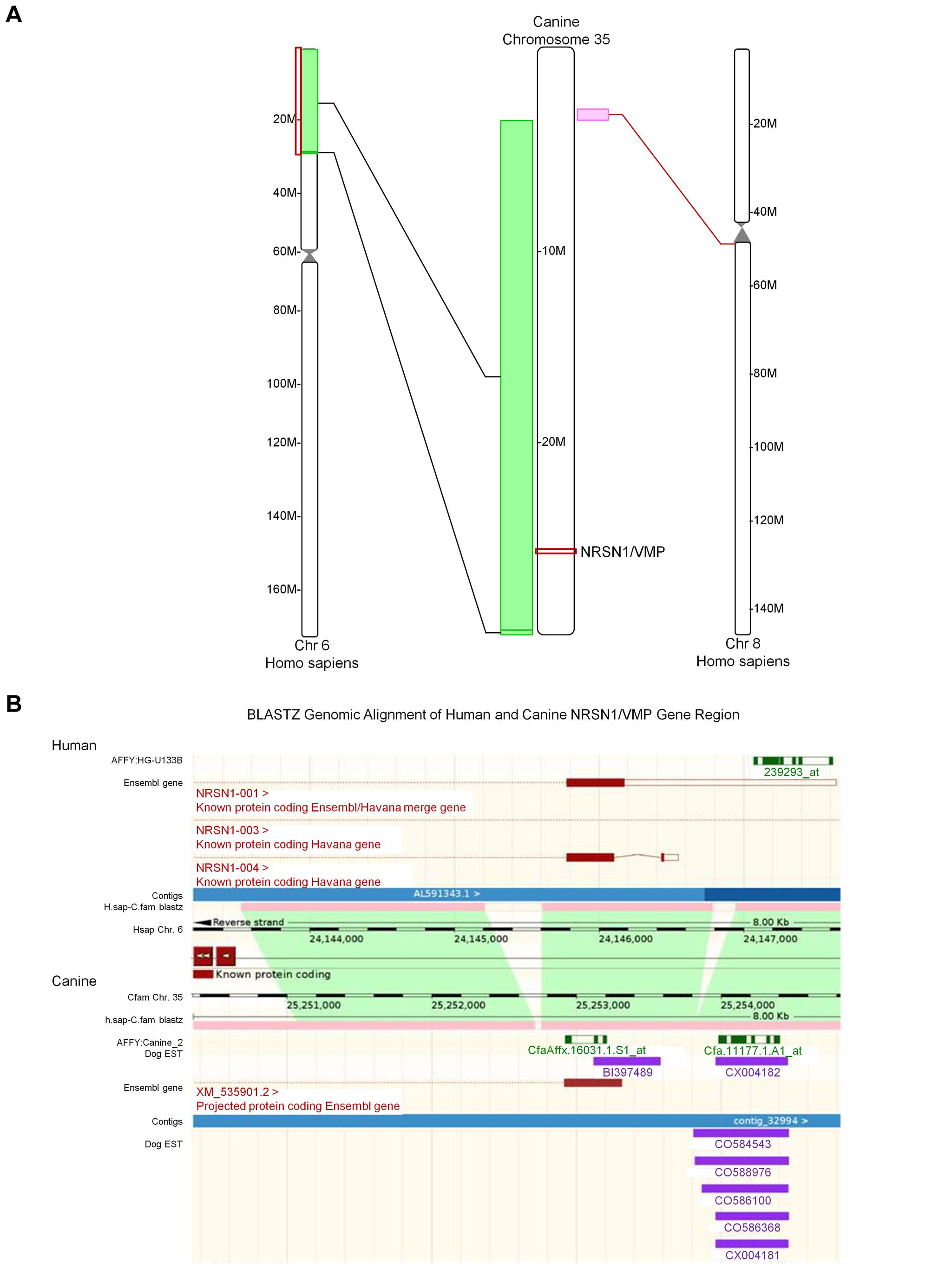

Supplement: Figure S6 — Resolution of transcript assignment for canine probesets mapping to the NRSN1 gene locus. A. Ensembl synteny map of canine chromosome 35 and human chromosome 6 highlighting the neurensin 1 (NRSN1) gene locus in each species. B. Ensembl BLASTZ genomic alignment of human chromosome 6 (top panel) and canine chromosome 35 (bottom panel) centered on the 3′ region of the NRSN1 gene locus. Affymetrix human GNFh probeset, 239293_at (NRSN1) and canine_2 probesets, CfaAffx.16031.1.S1_at (NRSN1), Cfa.11177.1.A1_at (unidentified), are aligned to their corresponding genomic regions. Canine EST evidence is shown in purple. (TIF) [file pone.0017107.s006.tif]
