# Supplementary material for: A Compendium of Canine Normal Tissue Gene Expression
Source: PLoS One. 2011 May 31;6(5):e17107. doi: 10.1371/journal.pone.0017107 (PMC3104984; doi:10.1371/journal.pone.0017107)
Supplement: Table S1 — Differentially expressed probesets and corresponding unique gene symbols in canine tissues. Describes the number of differentially expressed probesets and corresponding unique gene symbols for each of the ten canine organs examined in this dataset. (DOC) [file pone.0017107.s007.doc]

| **Canine Tissue** | **ANOVA Significant Canine Probesets (FDR= 0.001)** | **# Unique Gene Symbols** | **# Tissue Enriched**  **Probesets** | **# Unique Tissue**  **Enriched Gene**  **Symbols** | **# Tissue Selective Probesets** | **Percentage of Tissue Selective Probesets With No Gene Symbol** | **# Tissue Selective**  **Unique Gene Symbols** |
| --- | --- | --- | --- | --- | --- | --- | --- |
| Brain | 7944 | 4059 | 741 | 295 | 512 | 42% | 206 |
| Heart | 5803 | 2998 | 393 | 188 | 71 | 56% | 31 |
| Skeletal Muscle | 6289 | 3341 | 363 | 186 | 89 | 17% | 45 |
| Liver | 5300 | 2668 | 730 | 381 | 321 | 15% | 149 |
| Kidney | 4551 | 2312 | 464 | 226 | 154 | 24% | 63 |
| Jejunum | 3912 | 2148 | 359 | 222 | 134 | 15% | 84 |
| Pancreas | 10142 | 5590 | 188 | 101 | 85 | 12% | 43 |
| Spleen | 4114 | 2315 | 221 | 139 | 37 | 24% | 19 |
| Lymph  node | 7088 | 3901 | 239 | 148 | 24 | 29% | 11 |
| Lung | 4778 | 2513 | 263 | 146 | 75 | 24% | 39 |

Table S1: Differentially expressed probesets and corresponding unique gene symbols in canine tissues.

- ANOVA for all tissues (FDR, 0.001) resulted in 23070 significant probesets of 42860 total
