# Supplementary material for: A Compendium of Canine Normal Tissue Gene Expression
Source: PLoS One. 2011 May 31;6(5):e17107. doi: 10.1371/journal.pone.0017107 (PMC3104984; doi:10.1371/journal.pone.0017107)
Supplement: Table S2 — Canine lung selective probesets rank ordered with Fold Change vs. all tissues included. Defines probesets specific to the canine lung, and is an example of tissue selective ranking of probesets conducted for each of the ten canine organs examined in this dataset. (DOC) [file pone.0017107.s008.doc]

Table S2: Canine lung selective probesets rank ordered with Fold Change vs. all tissues included.

| **Probeset ID** | **Gene Symbol** | **Gene Title** | **Fold-Change (Lung vs. All)** | **p-value (Lung vs. All)** |
| --- | --- | --- | --- | --- |
| Cfa.12193.1.A1_at | --- | --- | 705.255 | 2.13E-29 |
| Cfa.12227.1.A1_at | SFTPC | surfactant protein C | 673.489 | 1.78E-23 |
| CfaAffx.15119.1.S1_at | SFTPC | surfactant protein C | 517.852 | 1.39E-26 |
| Cfa.12228.1.A1_s_at | SCGB1A1 | secretoglobin, family 1A, member 1 (uteroglobin) | 271.595 | 3.11E-27 |
| Cfa.3442.1.S1_s_at | AGER | advanced glycosylation end product-specific receptor | 161.716 | 1.42E-30 |
| CfaAffx.2089.1.S1_s_at | AGER | advanced glycosylation end product-specific receptor | 140.67 | 6.96E-30 |
| Cfa.12371.1.A1_s_at | FMO4 | flavin containing monooxygenase 4 | 122.248 | 8.64E-14 |
| Cfa.5149.1.A1_at | CLDN18 | claudin 18 | 101.678 | 7.27E-30 |
| Cfa.12371.1.A1_at | FMO4 | flavin containing monooxygenase 4 | 96.4518 | 7.20E-13 |
| CfaAffx.12336.1.S1_at | --- | --- | 92.3534 | 4.22E-33 |
| CfaAffx.8164.1.S1_at | --- | --- | 90.703 | 1.06E-32 |
| Cfa.12214.1.A1_at | CHI3L1 | chitinase 3-like 1 (cartilage glycoprotein-39) | 84.4318 | 3.67E-23 |
| CfaAffx.16068.1.S1_at | CHI3L1 | chitinase 3-like 1 (cartilage glycoprotein-39) | 61.8186 | 7.21E-31 |
| CfaAffx.22719.1.S1_at | CALCRL | calcitonin receptor-like | 61.7195 | 4.40E-13 |
| Cfa.661.1.A1_at | --- | --- | 55.8962 | 1.09E-15 |
| Cfa.18616.1.S1_at | TMEM100 | transmembrane protein 100 | 49.4084 | 1.10E-25 |
| Cfa.9510.1.A1_at | --- | --- | 49.257 | 7.56E-18 |
| CfaAffx.12092.1.S1_s_at | CLDN18 | claudin 18 | 47.163 | 4.03E-29 |
| Cfa.8884.1.A1_s_at | LOC483848 | similar to CG30418-PA | 44.7497 | 3.57E-17 |
| CfaAffx.29851.1.S1_at | CLIC3 | chloride intracellular channel 3 | 41.4575 | 3.22E-26 |
| CfaAffx.24150.1.S1_at | --- | --- | 39.8722 | 1.71E-27 |
| CfaAffx.21496.1.S1_s_at | SUSD2 | sushi domain containing 2 | 39.6824 | 8.56E-18 |
| CfaAffx.27421.1.S1_at | PMP22 | peripheral myelin protein 22 | 38.6838 | 4.74E-15 |
| Cfa.10039.1.A1_at | F2RL2 | coagulation factor II (thrombin) receptor-like 2 | 38.4792 | 1.62E-19 |
| Cfa.5688.1.A1_at | --- | --- | 29.9685 | 3.53E-13 |
| CfaAffx.30029.1.S1_at | CLIC2 | chloride intracellular channel 2 | 29.7628 | 1.89E-21 |
| Cfa.6002.1.S1_at | LOC448801 | mastin | 28.6612 | 7.60E-10 |
| CfaAffx.2273.1.S1_at | LOC612544 | similar to growth and transformation-dependent protein | 27.7624 | 8.50E-20 |
| Cfa.16308.1.S1_a_at | PMP22 | peripheral myelin protein 22 | 26.07 | 4.68E-12 |
| Cfa.16308.1.S1_s_at | PMP22 | peripheral myelin protein 22 | 25.7923 | 3.40E-13 |
| CfaAffx.26559.1.S1_s_at | TMEM100 | transmembrane protein 100 | 23.8446 | 2.43E-25 |
| CfaAffx.29953.1.S1_s_at | LOC448801 | mastin | 23.2324 | 4.20E-20 |
| CfaAffx.14120.1.S1_at | LOC609038 | similar to lysozyme | 21.8439 | 4.20E-16 |
| CfaAffx.14664.1.S1_s_at | F2RL2 | coagulation factor II (thrombin) receptor-like 2 | 21.4792 | 1.13E-24 |
| CfaAffx.4334.1.S1_s_at | OGN | osteoglycin | 21.1235 | 1.69E-08 |
| CfaAffx.10828.1.S1_at | ALOX5AP | arachidonate 5-lipoxygenase-activating protein | 20.8116 | 3.35E-12 |
| CfaAffx.5035.1.S1_s_at | GPNMB | glycoprotein (transmembrane) nmb | 20.0911 | 1.45E-10 |
| Cfa.17625.1.S1_at | EPAS1 | endothelial PAS domain protein 1 | 19.5746 | 8.23E-12 |
| CfaAffx.21807.1.S1_at | FZD2 | frizzled homolog 2 (Drosophila) | 18.9559 | 1.00E-15 |
| CfaAffx.11921.1.S1_s_at | PLUNC | palate, lung and nasal epithelium associated | 18.9188 | 8.43E-09 |
| CfaAffx.10828.1.S1_s_at | ALOX5AP | arachidonate 5-lipoxygenase-activating protein | 18.8493 | 3.96E-16 |
| Cfa.9431.1.A1_at | --- | --- | 17.957 | 5.08E-16 |
| Cfa.12129.1.A1_at | PLUNC | palate, lung and nasal epithelium associated | 17.9255 | 3.28E-09 |
| Cfa.13689.1.A1_at | --- | --- | 17.7038 | 1.59E-22 |
| Cfa.90.1.A1_at | PPL | periplakin | 17.2303 | 1.32E-13 |
| Cfa.533.1.S1_at | --- | --- | 17.0534 | 5.03E-08 |
| Cfa.6867.1.A1_at | DARC | Duffy blood group, chemokine receptor | 16.8433 | 2.69E-10 |
| CfaAffx.17502.1.S1_at | AXIN2 | axin 2 | 16.4429 | 1.71E-11 |
| CfaAffx.1239.1.S1_s_at | LOC476202 | similar to connective tissue growth factor | 16.3998 | 2.68E-13 |
| Cfa.17108.1.S1_at | MSLN | mesothelin | 16.3943 | 3.20E-15 |
| Cfa.16947.1.A1_at | CH25H | cholesterol 25-hydroxylase | 16.1316 | 2.64E-09 |
| Cfa.9196.1.A1_at | --- | --- | 15.8087 | 2.55E-13 |
| Cfa.11304.1.A1_at | TPBG | trophoblast glycoprotein | 15.3433 | 5.14E-11 |
| Cfa.4845.1.A1_at | THSD1 | thrombospondin, type I, domain containing 1 | 15.3138 | 1.17E-12 |
| CfaAffx.1589.1.S1_at | --- | --- | 15.3137 | 1.05E-26 |
| Cfa.15435.1.A1_at | --- | --- | 15.3057 | 1.84E-17 |
| CfaAffx.30577.1.S1_at | HSD17B2 | hydroxysteroid (17-beta) dehydrogenase 2 | 15.029 | 6.44E-20 |
| Cfa.15350.1.A1_at | LOC480468 | hypothetical LOC480468 | 14.7568 | 5.24E-13 |
| Cfa.2608.1.A1_at | --- | --- | 14.505 | 8.65E-13 |
| CfaAffx.11921.1.S1_at | PLUNC | palate, lung and nasal epithelium associated | 14.1092 | 4.87E-08 |
| CfaAffx.14170.1.S1_at | SCD5 | stearoyl-CoA desaturase 5 | 12.6553 | 8.13E-15 |
| CfaAffx.1666.1.S1_at | SFTA2 | surfactant associated 2 | 12.5552 | 4.08E-18 |
| Cfa.306.1.S1_at | --- | --- | 12.1925 | 1.20E-11 |
| Cfa.21301.1.S1_at | --- | --- | 11.7297 | 5.65E-16 |
| CfaAffx.14553.1.S1_at | AMIGO2 | adhesion molecule with Ig-like domain 2 | 11.2815 | 1.18E-24 |
| Cfa.11315.1.A1_at | --- | --- | 11.232 | 2.86E-16 |
| Cfa.6457.1.S1_at | LOC477194 | similar to Long palate, lung and nasal epithelium carcinoma associated protein 1 precursor (Von Ebner minor salivary gland protein) (VEMSGP) | 11.1466 | 2.48E-08 |
| Cfa.6727.1.A1_at | LOC477964 | hypothetical LOC477964 | 11.0902 | 4.88E-15 |
| Cfa.19766.1.A1_at | --- | --- | 11.008 | 1.17E-10 |
| Cfa.21578.1.S1_at | TMEM2 | transmembrane protein 2 | 10.6933 | 2.66E-15 |
| CfaAffx.27870.1.S1_s_at | MFAP4 | microfibrillar-associated protein 4 | 10.6886 | 9.60E-16 |
| Cfa.18423.1.S1_at | SNCG | synuclein, gamma (breast cancer-specific protein 1) | 10.6041 | 1.30E-08 |
| Cfa.125.1.S1_s_at | EDN1 | endothelin 1 | 10.3445 | 6.36E-13 |
| Cfa.4590.1.S1_s_at | ASPN | asporin | 10.3431 | 7.95E-07 |
| CfaAffx.4016.1.S1_s_at | GPR116 | G protein-coupled receptor 116 | 10.0712 | 1.07E-08 |
