# Supplementary material for: A Compendium of Canine Normal Tissue Gene Expression
Source: PLoS One. 2011 May 31;6(5):e17107. doi: 10.1371/journal.pone.0017107 (PMC3104984; doi:10.1371/journal.pone.0017107)
Supplement: Table S3 — Canine Brain Selective Probesets Over Represented GO Terms. Defines over-represented GO terms in the canine brain, and is an example of the process used to define GO terms for each of the ten canine organs examined in this dataset. (DOC) [file pone.0017107.s009.doc]

Table S3: Canine Brain Selective Probesets Over Represented GO Terms

| **GO** | **NAME** | **FDR** | **FWER** | **p-Value** | **#Test** | **#Ref** | **#not**  **AnnotTest** | **#not**  **AnnotRef** | **Over/**  **Under** |
| --- | --- | --- | --- | --- | --- | --- | --- | --- | --- |
| GO:0007275 | multicellular organismal development | 2.10E-08 | 3.63E-08 | 0 | 56 | 3252 | 86 | 17716 | over |
| GO:0022008 | neurogenesis | 2.10E-08 | 3.64E-08 | 1.03E-12 | 29 | 661 | 113 | 20307 | over |
| GO:0048856 | anatomical structure development | 2.10E-08 | 3.68E-08 | 3.04E-12 | 54 | 2797 | 88 | 18171 | over |
| GO:0048699 | generation of neurons | 2.10E-08 | 3.68E-08 | 3.29E-12 | 27 | 623 | 115 | 20345 | over |
| GO:0007399 | nervous system development | 2.10E-08 | 4.08E-08 | 1.76E-11 | 47 | 1228 | 95 | 19740 | over |
| GO:0048731 | system development | 2.10E-08 | 4.10E-08 | 1.95E-11 | 52 | 2630 | 90 | 18338 | over |
| GO:0048468 | cell development | 2.10E-08 | 4.14E-08 | 2.61E-11 | 30 | 961 | 112 | 20007 | over |
| GO:0032501 | multicellular organismal process | 2.10E-08 | 4.21E-08 | 3.23E-11 | 71 | 4995 | 71 | 15973 | over |
| GO:0042995 | cell projection | 2.14E-08 | 5.30E-08 | 6.17E-11 | 26 | 809 | 116 | 20159 | over |
| GO:0032502 | developmental process | 2.14E-08 | 5.36E-08 | 6.41E-11 | 60 | 3858 | 82 | 17110 | over |
| GO:0007268 | synaptic transmission | 5.97E-08 | 1.64E-07 | 4.07E-10 | 18 | 397 | 124 | 20571 | over |
| GO:0019226 | transmission of nerve impulse | 7.22E-08 | 2.18E-07 | 5.76E-10 | 19 | 460 | 123 | 20508 | over |
| GO:0043005 | neuron projection | 7.22E-08 | 2.66E-07 | 5.97E-10 | 17 | 356 | 125 | 20612 | over |
| GO:0048869 | cellular developmental process | 7.22E-08 | 2.71E-07 | 6.35E-10 | 35 | 1608 | 107 | 19360 | over |
| GO:0030154 | cell differentiation | 7.22E-08 | 2.71E-07 | 6.35E-10 | 35 | 1608 | 107 | 19360 | over |
| GO:0030182 | neuron differentiation | 1.07E-07 | 4.30E-07 | 1.30E-09 | 19 | 484 | 123 | 20484 | over |
| GO:0043025 | cell soma | 7.56E-07 | 3.21E-06 | 9.39E-09 | 12 | 185 | 130 | 20783 | over |
| GO:0048666 | neuron development | 1.14E-06 | 5.14E-06 | 1.10E-08 | 16 | 380 | 126 | 20588 | over |
| GO:0016020 | membrane | 1.20E-06 | 5.69E-06 | 1.35E-08 | 90 | 8344 | 52 | 12624 | over |
| GO:0045202 | synapse | 1.49E-06 | 7.47E-06 | 1.98E-08 | 16 | 397 | 126 | 20571 | over |
| GO:0030424 | axon | 1.91E-06 | 1.00E-05 | 2.72E-08 | 11 | 163 | 131 | 20805 | over |
| GO:0044456 | synapse part | 2.57E-06 | 1.42E-05 | 3.38E-08 | 13 | 255 | 129 | 20713 | over |
| GO:0031175 | neurite development | 2.79E-06 | 1.60E-05 | 3.72E-08 | 14 | 307 | 128 | 20661 | over |
| GO:0005519 | cytoskeletal regulatory protein binding | 4.90E-06 | 2.94E-05 | 6.76E-08 | 4 | 3 | 138 | 20965 | over |
| GO:0050877 | neurological system process | 6.05E-06 | 3.78E-05 | 1.00E-07 | 32 | 1689 | 110 | 19279 | over |
| GO:0007267 | cell-cell signaling | 1.23E-05 | 8.00E-05 | 2.18E-07 | 20 | 747 | 122 | 20221 | over |
| GO:0051969 | regulation of transmission of nerve impulse | 1.25E-05 | 8.41E-05 | 2.34E-07 | 10 | 160 | 132 | 20808 | over |
| GO:0030426 | growth cone | 2.84E-05 | 1.99E-04 | 3.33E-07 | 7 | 60 | 135 | 20908 | over |
| GO:0030427 | site of polarized growth | 2.85E-05 | 2.07E-04 | 3.70E-07 | 7 | 61 | 135 | 20907 | over |
| GO:0031644 | regulation of neurological system process | 3.32E-05 | 2.49E-04 | 4.63E-07 | 10 | 173 | 132 | 20795 | over |
| GO:0005886 | plasma membrane | 4.20E-05 | 3.26E-04 | 6.47E-07 | 53 | 4083 | 89 | 16885 | over |
| GO:0005515 | protein binding | 9.49E-05 | 7.87E-04 | 1.51E-06 | 91 | 9259 | 51 | 11709 | over |
| GO:0050804 | regulation of synaptic transmission | 9.49E-05 | 7.91E-04 | 1.54E-06 | 9 | 153 | 133 | 20815 | over |
| GO:0030030 | cell projection organization | 9.49E-05 | 8.29E-04 | 1.68E-06 | 14 | 426 | 128 | 20542 | over |
| GO:0003008 | system process | 9.49E-05 | 8.30E-04 | 1.71E-06 | 33 | 2021 | 109 | 18947 | over |
| GO:0051129 | negative regulation of cellular component organization | 1.19E-04 | 0.00106741 | 2.49E-06 | 8 | 120 | 134 | 20848 | over |
| GO:0006810 | transport | 1.27E-04 | 0.00117298 | 2.63E-06 | 48 | 3683 | 94 | 17285 | over |
| GO:0051234 | establishment of localization | 1.72E-04 | 0.00163204 | 3.17E-06 | 48 | 3707 | 94 | 17261 | over |
| GO:0007026 | negative regulation of microtubule depolymerization | 1.78E-04 | 0.00173095 | 3.35E-06 | 4 | 12 | 138 | 20956 | over |
| GO:0007611 | learning or memory | 1.78E-04 | 0.00178012 | 3.51E-06 | 8 | 126 | 134 | 20842 | over |
| GO:0007409 | axonogenesis | 1.92E-04 | 0.00196887 | 4.28E-06 | 10 | 224 | 132 | 20744 | over |
| GO:0051179 | localization | 2.11E-04 | 0.00221625 | 4.93E-06 | 52 | 4238 | 90 | 16730 | over |
| GO:0031114 | regulation of microtubule depolymerization | 2.31E-04 | 0.00248411 | 5.58E-06 | 4 | 14 | 138 | 20954 | over |
| GO:0031111 | negative regulation of microtubule polymerization or depolymerization | 3.63E-04 | 0.00398836 | 8.74E-06 | 4 | 16 | 138 | 20952 | over |
| GO:0031344 | regulation of cell projection organization | 3.74E-04 | 0.0042033 | 9.60E-06 | 7 | 103 | 135 | 20865 | over |
| GO:0007610 | behavior | 4.26E-04 | 0.00515317 | 1.05E-05 | 14 | 502 | 128 | 20466 | over |
| GO:0048167 | regulation of synaptic plasticity | 4.26E-04 | 0.00519449 | 1.06E-05 | 6 | 68 | 136 | 20900 | over |
| GO:0048667 | cell morphogenesis involved in neuron differentiation | 4.26E-04 | 0.00541638 | 1.08E-05 | 10 | 250 | 132 | 20718 | over |
| GO:0048858 | cell projection morphogenesis | 4.26E-04 | 0.00541638 | 1.08E-05 | 10 | 250 | 132 | 20718 | over |
| GO:0032990 | cell part morphogenesis | 4.26E-04 | 0.00541638 | 1.08E-05 | 10 | 250 | 132 | 20718 | over |
| GO:0048812 | neurite morphogenesis | 4.26E-04 | 0.00541638 | 1.08E-05 | 10 | 250 | 132 | 20718 | over |
| GO:0050770 | regulation of axonogenesis | 4.92E-04 | 0.00638059 | 1.44E-05 | 6 | 72 | 136 | 20896 | over |
| GO:0000904 | cell morphogenesis involved in differentiation | 6.79E-04 | 0.00895902 | 1.99E-05 | 10 | 269 | 132 | 20699 | over |
| GO:0000902 | cell morphogenesis | 6.81E-04 | 0.00914681 | 2.09E-05 | 14 | 535 | 128 | 20433 | over |
| GO:0010769 | regulation of cell morphogenesis involved in differentiation | 8.99E-04 | 0.0122913 | 2.69E-05 | 6 | 81 | 136 | 20887 | over |
| GO:0022836 | gated channel activity | 9.82E-04 | 0.0136505 | 3.23E-05 | 11 | 348 | 131 | 20620 | over |
| GO:0031110 | regulation of microtubule polymerization or depolymerization | 0.00110347 | 0.0162225 | 3.54E-05 | 4 | 24 | 138 | 20944 | over |
| GO:0070507 | regulation of microtubule cytoskeleton organization | 0.00110347 | 0.0162225 | 3.54E-05 | 4 | 24 | 138 | 20944 | over |
| GO:0045211 | postsynaptic membrane | 0.00110347 | 0.0163818 | 3.63E-05 | 7 | 128 | 135 | 20840 | over |
| GO:0044057 | regulation of system process | 0.00110347 | 0.0164159 | 3.67E-05 | 11 | 353 | 131 | 20615 | over |
| GO:0015267 | channel activity | 0.00117609 | 0.0180643 | 4.36E-05 | 13 | 499 | 129 | 20469 | over |
| GO:0022803 | passive transmembrane transporter activity | 0.00117609 | 0.0180643 | 4.36E-05 | 13 | 499 | 129 | 20469 | over |
| GO:0033269 | internode region of axon | 0.00201773 | 0.0327446 | 4.49E-05 | 2 | 0 | 140 | 20968 | over |
| GO:0031233 | intrinsic to external side of plasma membrane | 0.00201773 | 0.0327446 | 4.49E-05 | 2 | 0 | 140 | 20968 | over |
| GO:0031362 | anchored to external side of plasma membrane | 0.00201773 | 0.0327446 | 4.49E-05 | 2 | 0 | 140 | 20968 | over |
| GO:0047617 | acyl-CoA hydrolase activity | 0.00201773 | 0.0327446 | 4.49E-05 | 2 | 0 | 140 | 20968 | over |
| GO:0050771 | negative regulation of axonogenesis | 0.00202737 | 0.0333885 | 4.69E-05 | 4 | 26 | 138 | 20942 | over |
| GO:0007158 | neuron adhesion | 0.00212075 | 0.0354167 | 4.73E-05 | 3 | 8 | 139 | 20960 | over |
| GO:0007154 | cell communication | 0.00212075 | 0.035922 | 4.93E-05 | 57 | 5221 | 85 | 15747 | over |
| GO:0030425 | dendrite | 0.00218516 | 0.0375185 | 5.56E-05 | 8 | 188 | 134 | 20780 | over |
| GO:0045921 | positive regulation of exocytosis | 0.00239688 | 0.0418989 | 6.27E-05 | 3 | 9 | 139 | 20959 | over |
| GO:0042391 | regulation of membrane potential | 0.00239688 | 0.0427554 | 6.80E-05 | 7 | 142 | 135 | 20826 | over |
| GO:0015630 | microtubule cytoskeleton | 0.00239688 | 0.042945 | 6.90E-05 | 14 | 599 | 128 | 20369 | over |
| GO:0031345 | negative regulation of cell projection organization | 0.00239688 | 0.0433738 | 6.90E-05 | 4 | 29 | 138 | 20939 | over |
| GO:0032268 | regulation of cellular protein metabolic process | 0.00249451 | 0.0456953 | 7.78E-05 | 14 | 606 | 128 | 20362 | over |
| GO:0044459 | plasma membrane part | 0.00262739 | 0.0486951 | 8.25E-05 | 35 | 2663 | 107 | 18305 | over |
| GO:0007417 | central nervous system development | 0.0026308 | 0.0493823 | 8.73E-05 | 12 | 461 | 130 | 20507 | over |
| GO:0005216 | ion channel activity | 0.00275441 | 0.0522944 | 1.00E-04 | 12 | 468 | 130 | 20500 | over |
| GO:0007612 | learning | 0.00276083 | 0.0530667 | 1.02E-04 | 5 | 64 | 137 | 20904 | over |
| GO:0044430 | cytoskeletal part | 0.0028899 | 0.0561597 | 1.03E-04 | 19 | 1051 | 123 | 19917 | over |
| GO:0032886 | regulation of microtubule-based process | 0.00292923 | 0.057764 | 1.09E-04 | 4 | 33 | 138 | 20935 | over |
| GO:0045664 | regulation of neuron differentiation | 0.00292923 | 0.0582821 | 1.11E-04 | 7 | 154 | 135 | 20814 | over |
| GO:0007017 | microtubule-based process | 0.00294983 | 0.0593739 | 1.18E-04 | 10 | 335 | 132 | 20633 | over |
| GO:0060219 | camera-type eye photoreceptor cell differentiation | 0.00456535 | 0.0955596 | 1.34E-04 | 2 | 1 | 140 | 20967 | over |
| GO:0048151 | hyperphosphorylation | 0.00456535 | 0.0955596 | 1.34E-04 | 2 | 1 | 140 | 20967 | over |
| GO:0042670 | retinal cone cell differentiation | 0.00456535 | 0.0955596 | 1.34E-04 | 2 | 1 | 140 | 20967 | over |
| GO:0045917 | positive regulation of complement activation | 0.00456535 | 0.0955596 | 1.34E-04 | 2 | 1 | 140 | 20967 | over |
| GO:0046549 | retinal cone cell development | 0.00456535 | 0.0955596 | 1.34E-04 | 2 | 1 | 140 | 20967 | over |
| GO:0022838 | substrate specific channel activity | 0.00458871 | 0.0970607 | 1.36E-04 | 12 | 484 | 130 | 20484 | over |
| GO:0007214 | gamma-aminobutyric acid signaling pathway | 0.00515683 | 0.109552 | 1.80E-04 | 4 | 38 | 138 | 20930 | over |
| GO:0005737 | cytoplasm | 0.00549445 | 0.117503 | 2.00E-04 | 77 | 8204 | 65 | 12764 | over |
| GO:0051246 | regulation of protein metabolic process | 0.00553843 | 0.119647 | 2.13E-04 | 14 | 669 | 128 | 20299 | over |
| GO:0005246 | calcium channel regulator activity | 0.00553843 | 0.120824 | 2.16E-04 | 4 | 40 | 138 | 20928 | over |
| GO:0010721 | negative regulation of cell development | 0.00567823 | 0.128331 | 2.36E-04 | 4 | 41 | 138 | 20927 | over |
| GO:0017157 | regulation of exocytosis | 0.00567823 | 0.128331 | 2.36E-04 | 4 | 41 | 138 | 20927 | over |
| GO:0050768 | negative regulation of neurogenesis | 0.00567823 | 0.128331 | 2.36E-04 | 4 | 41 | 138 | 20927 | over |
| GO:0005261 | cation channel activity | 0.00567823 | 0.129071 | 2.41E-04 | 10 | 367 | 132 | 20601 | over |
| GO:0051239 | regulation of multicellular organismal process | 0.00567823 | 0.130265 | 2.50E-04 | 17 | 942 | 125 | 20026 | over |
| GO:0005856 | cytoskeleton | 0.00567823 | 0.13111 | 2.55E-04 | 23 | 1521 | 119 | 19447 | over |
| GO:0043209 | myelin sheath | 0.00594757 | 0.138165 | 2.67E-04 | 3 | 16 | 139 | 20952 | over |
| GO:0051281 | positive regulation of release of sequestered calcium ion into cytosol | 0.00726102 | 0.174299 | 2.67E-04 | 2 | 2 | 140 | 20966 | over |
| GO:0019911 | structural constituent of myelin sheath | 0.00726102 | 0.174299 | 2.67E-04 | 2 | 2 | 140 | 20966 | over |
| GO:0010524 | positive regulation of calcium ion transport into cytosol | 0.00726102 | 0.174299 | 2.67E-04 | 2 | 2 | 140 | 20966 | over |
| GO:0009987 | cellular process | 0.00726102 | 0.174587 | 2.69E-04 | 113 | 13825 | 29 | 7143 | over |
| GO:0005626 | insoluble fraction | 0.00726102 | 0.175041 | 2.72E-04 | 18 | 1041 | 124 | 19927 | over |
| GO:0010646 | regulation of cell communication | 0.00726102 | 0.175041 | 2.72E-04 | 18 | 1041 | 124 | 19927 | over |
| GO:0046873 | metal ion transmembrane transporter activity | 0.00731066 | 0.178086 | 2.88E-04 | 11 | 449 | 131 | 20519 | over |
| GO:0032101 | regulation of response to external stimulus | 0.00731066 | 0.179131 | 2.95E-04 | 6 | 128 | 136 | 20840 | over |
| GO:0051128 | regulation of cellular component organization | 0.00761794 | 0.187463 | 3.14E-04 | 13 | 612 | 129 | 20356 | over |
| GO:0005875 | microtubule associated complex | 0.0082589 | 0.203179 | 3.81E-04 | 7 | 190 | 135 | 20778 | over |
| GO:0051050 | positive regulation of transport | 0.00833393 | 0.20773 | 4.03E-04 | 6 | 136 | 136 | 20832 | over |
| GO:0050767 | regulation of neurogenesis | 0.00833393 | 0.208128 | 4.05E-04 | 7 | 192 | 135 | 20776 | over |
| GO:0031988 | membrane-bounded vesicle | 0.00865395 | 0.216889 | 4.32E-04 | 12 | 551 | 130 | 20417 | over |
| GO:0006813 | potassium ion transport | 0.00869954 | 0.219596 | 4.43E-04 | 7 | 195 | 135 | 20773 | over |
| GO:0048156 | tau protein binding | 0.0102505 | 0.25716 | 4.43E-04 | 2 | 3 | 140 | 20965 | over |
| GO:0002922 | positive regulation of humoral immune response | 0.0102505 | 0.25716 | 4.43E-04 | 2 | 3 | 140 | 20965 | over |
| GO:0001578 | microtubule bundle formation | 0.0106107 | 0.266828 | 4.78E-04 | 3 | 20 | 139 | 20948 | over |
| GO:0005624 | membrane fraction | 0.0109731 | 0.276546 | 5.43E-04 | 17 | 1009 | 125 | 19959 | over |
| GO:0050794 | regulation of cellular process | 0.0112403 | 0.285221 | 5.54E-04 | 80 | 8873 | 62 | 12095 | over |
| GO:0051960 | regulation of nervous system development | 0.0112403 | 0.286248 | 5.58E-04 | 7 | 203 | 135 | 20765 | over |
| GO:0043242 | negative regulation of protein complex disassembly | 0.0113193 | 0.28995 | 5.88E-04 | 4 | 53 | 138 | 20915 | over |
| GO:0060284 | regulation of cell development | 0.0113554 | 0.292737 | 6.07E-04 | 7 | 206 | 135 | 20762 | over |
| GO:0033267 | axon part | 0.0116849 | 0.301853 | 6.28E-04 | 4 | 54 | 138 | 20914 | over |
| GO:0034235 | GPI anchor binding | 0.0134897 | 0.347237 | 6.62E-04 | 2 | 4 | 140 | 20964 | over |
| GO:0050858 | negative regulation of antigen receptor-mediated signaling pathway | 0.0134897 | 0.347237 | 6.62E-04 | 2 | 4 | 140 | 20964 | over |
| GO:0050860 | negative regulation of T cell receptor signaling pathway | 0.0134897 | 0.347237 | 6.62E-04 | 2 | 4 | 140 | 20964 | over |
| GO:0008021 | synaptic vesicle | 0.0134897 | 0.348397 | 6.62E-04 | 5 | 98 | 137 | 20870 | over |
| GO:0051493 | regulation of cytoskeleton organization | 0.0139477 | 0.362712 | 7.23E-04 | 5 | 100 | 137 | 20868 | over |
| GO:0031225 | anchored to membrane | 0.0139477 | 0.362712 | 7.23E-04 | 5 | 100 | 137 | 20868 | over |
| GO:0006873 | cellular ion homeostasis | 0.0139477 | 0.364487 | 7.40E-04 | 9 | 350 | 133 | 20618 | over |
| GO:0044425 | membrane part | 0.0140698 | 0.369226 | 7.66E-04 | 64 | 6701 | 78 | 14267 | over |
| GO:0030001 | metal ion transport | 0.0142374 | 0.378823 | 7.89E-04 | 14 | 765 | 128 | 20203 | over |
| GO:0005244 | voltage-gated ion channel activity | 0.0142374 | 0.379343 | 7.96E-04 | 7 | 216 | 135 | 20752 | over |
| GO:0022832 | voltage-gated channel activity | 0.0142374 | 0.379343 | 7.96E-04 | 7 | 216 | 135 | 20752 | over |
| GO:0050905 | neuromuscular process | 0.0146706 | 0.390526 | 8.59E-04 | 4 | 59 | 138 | 20909 | over |
| GO:0005267 | potassium channel activity | 0.0149121 | 0.398739 | 8.88E-04 | 6 | 159 | 136 | 20809 | over |
| GO:0034702 | ion channel complex | 0.0149121 | 0.399965 | 9.06E-04 | 7 | 221 | 135 | 20747 | over |
| GO:0055082 | cellular chemical homeostasis | 0.0149338 | 0.402646 | 9.16E-04 | 9 | 361 | 133 | 20607 | over |
| GO:0030449 | regulation of complement activation | 0.0166164 | 0.44332 | 9.23E-04 | 2 | 5 | 140 | 20963 | over |
| GO:0010522 | regulation of calcium ion transport into cytosol | 0.0166164 | 0.44332 | 9.23E-04 | 2 | 5 | 140 | 20963 | over |
| GO:0051279 | regulation of release of sequestered calcium ion into cytosol | 0.0166164 | 0.44332 | 9.23E-04 | 2 | 5 | 140 | 20963 | over |
| GO:0043062 | extracellular structure organization | 0.0172259 | 0.457497 | 0.00100499 | 6 | 163 | 136 | 20805 | over |
| GO:0051047 | positive regulation of secretion | 0.0173001 | 0.46126 | 0.0010237 | 4 | 62 | 138 | 20906 | over |
| GO:0010608 | posttranscriptional regulation of gene expression | 0.017331 | 0.464181 | 0.00104724 | 8 | 295 | 134 | 20673 | over |
| GO:0048169 | regulation of long-term neuronal synaptic plasticity | 0.0175119 | 0.469987 | 0.00105907 | 3 | 27 | 139 | 20941 | over |
| GO:0005215 | transporter activity | 0.0178441 | 0.478663 | 0.0011481 | 23 | 1698 | 119 | 19270 | over |
| GO:0051494 | negative regulation of cytoskeleton organization | 0.0182758 | 0.490471 | 0.00120899 | 4 | 65 | 138 | 20903 | over |
| GO:0016023 | cytoplasmic membrane-bounded vesicle | 0.0182758 | 0.491484 | 0.0012196 | 11 | 537 | 131 | 20431 | over |
| GO:0048143 | astrocyte activation | 0.02019 | 0.530625 | 0.00122512 | 2 | 6 | 140 | 20962 | over |
| GO:0045596 | negative regulation of cell differentiation | 0.02019 | 0.531843 | 0.00123847 | 6 | 170 | 136 | 20798 | over |
| GO:0005874 | microtubule | 0.02019 | 0.533379 | 0.00126202 | 8 | 304 | 134 | 20664 | over |
| GO:0016917 | GABA receptor activity | 0.020405 | 0.541851 | 0.00128119 | 3 | 29 | 139 | 20939 | over |
| GO:0048306 | calcium-dependent protein binding | 0.020405 | 0.541851 | 0.00128119 | 3 | 29 | 139 | 20939 | over |
| GO:0007628 | adult walking behavior | 0.0208528 | 0.553892 | 0.00140241 | 3 | 30 | 139 | 20938 | over |
| GO:0008289 | lipid binding | 0.0208528 | 0.554306 | 0.00140839 | 11 | 547 | 131 | 20421 | over |
| GO:0006417 | regulation of translation | 0.0212257 | 0.563026 | 0.00150615 | 7 | 242 | 135 | 20726 | over |
| GO:0008092 | cytoskeletal protein binding | 0.021548 | 0.570804 | 0.00153851 | 12 | 640 | 130 | 20328 | over |
| GO:0045576 | mast cell activation | 0.0235692 | 0.611509 | 0.00156821 | 2 | 7 | 140 | 20961 | over |
| GO:0002920 | regulation of humoral immune response | 0.0235692 | 0.611509 | 0.00156821 | 2 | 7 | 140 | 20961 | over |
| GO:0000267 | cell fraction | 0.0235692 | 0.612664 | 0.00159765 | 19 | 1318 | 123 | 19650 | over |
| GO:0050801 | ion homeostasis | 0.0235692 | 0.612781 | 0.00159865 | 9 | 392 | 133 | 20576 | over |
| GO:0008066 | glutamate receptor activity | 0.0239368 | 0.620995 | 0.0016659 | 3 | 32 | 139 | 20936 | over |
| GO:0031982 | vesicle | 0.0239368 | 0.623009 | 0.00170374 | 12 | 648 | 130 | 20320 | over |
| GO:0031224 | intrinsic to membrane | 0.0240027 | 0.627772 | 0.00176596 | 56 | 5817 | 86 | 15151 | over |
| GO:0050789 | regulation of biological process | 0.0240027 | 0.628507 | 0.00178157 | 80 | 9174 | 62 | 11794 | over |
| GO:0030054 | cell junction | 0.0248801 | 0.643938 | 0.0019367 | 10 | 485 | 132 | 20483 | over |
| GO:0048147 | negative regulation of fibroblast proliferation | 0.0262669 | 0.674717 | 0.00195162 | 2 | 8 | 140 | 20960 | over |
| GO:0006378 | mRNA polyadenylation | 0.0262669 | 0.674717 | 0.00195162 | 2 | 8 | 140 | 20960 | over |
| GO:0016944 | RNA polymerase II transcription elongation factor activity | 0.0262669 | 0.674717 | 0.00195162 | 2 | 8 | 140 | 20960 | over |
| GO:0016524 | latrotoxin receptor activity | 0.0262669 | 0.674717 | 0.00195162 | 2 | 8 | 140 | 20960 | over |
| GO:0048154 | S100 beta binding | 0.0262669 | 0.674717 | 0.00195162 | 2 | 8 | 140 | 20960 | over |
| GO:0022843 | voltage-gated cation channel activity | 0.0271338 | 0.688674 | 0.00208311 | 6 | 189 | 136 | 20779 | over |
| GO:0034703 | cation channel complex | 0.0280739 | 0.703104 | 0.00224862 | 6 | 192 | 136 | 20776 | over |
| GO:0060042 | retina morphogenesis in camera-type eye | 0.0310065 | 0.742802 | 0.00237481 | 2 | 9 | 140 | 20959 | over |
| GO:0043631 | RNA polyadenylation | 0.0310065 | 0.742802 | 0.00237481 | 2 | 9 | 140 | 20959 | over |
| GO:0010639 | negative regulation of organelle organization | 0.0310065 | 0.744494 | 0.00239034 | 4 | 79 | 138 | 20889 | over |
| GO:0007270 | nerve-nerve synaptic transmission | 0.0312551 | 0.750037 | 0.00245389 | 3 | 37 | 139 | 20931 | over |
| GO:0005230 | extracellular ligand-gated ion channel activity | 0.0312551 | 0.752669 | 0.00249693 | 4 | 80 | 138 | 20888 | over |
| GO:0065007 | biological regulation | 0.0312551 | 0.753132 | 0.00251035 | 83 | 9709 | 59 | 11259 | over |
| GO:0033043 | regulation of organelle organization | 0.031436 | 0.757041 | 0.00258837 | 5 | 135 | 137 | 20833 | over |
| GO:0007411 | axon guidance | 0.0317004 | 0.764143 | 0.00266927 | 5 | 136 | 137 | 20832 | over |
| GO:0005509 | calcium ion binding | 0.0317004 | 0.766033 | 0.00272083 | 15 | 973 | 127 | 19995 | over |
| GO:0043244 | regulation of protein complex disassembly | 0.0317004 | 0.767026 | 0.00275198 | 5 | 137 | 137 | 20831 | over |
| GO:0022604 | regulation of cell morphogenesis | 0.0317004 | 0.76741 | 0.00277161 | 8 | 346 | 134 | 20622 | over |
| GO:0032846 | positive regulation of homeostatic process | 0.0338756 | 0.79577 | 0.00283723 | 2 | 10 | 140 | 20958 | over |
| GO:0050856 | regulation of T cell receptor signaling pathway | 0.0338756 | 0.79577 | 0.00283723 | 2 | 10 | 140 | 20958 | over |
| GO:0016290 | palmitoyl-CoA hydrolase activity | 0.0338756 | 0.79577 | 0.00283723 | 2 | 10 | 140 | 20958 | over |
| GO:0044463 | cell projection part | 0.0338756 | 0.798548 | 0.00294152 | 6 | 203 | 136 | 20765 | over |
| GO:0030534 | adult behavior | 0.0338756 | 0.799279 | 0.00295597 | 4 | 84 | 138 | 20884 | over |
| GO:0019725 | cellular homeostasis | 0.0338756 | 0.799994 | 0.00298416 | 9 | 431 | 133 | 20537 | over |
| GO:0051924 | regulation of calcium ion transport | 0.0342082 | 0.804807 | 0.00302044 | 3 | 40 | 139 | 20928 | over |
| GO:0045595 | regulation of cell differentiation | 0.0354515 | 0.817687 | 0.00331138 | 9 | 438 | 133 | 20530 | over |
| GO:0008889 | glycerophosphodiester phosphodiesterase activity | 0.0368618 | 0.832738 | 0.00333834 | 2 | 11 | 140 | 20957 | over |
| GO:0016291 | acyl-CoA thioesterase activity | 0.0368618 | 0.832738 | 0.00333834 | 2 | 11 | 140 | 20957 | over |
| GO:0022857 | transmembrane transporter activity | 0.037798 | 0.841671 | 0.00364298 | 17 | 1209 | 125 | 19759 | over |
| GO:0007613 | memory | 0.038022 | 0.844873 | 0.00366118 | 3 | 43 | 139 | 20925 | over |
| GO:0032940 | secretion by cell | 0.0382377 | 0.847966 | 0.00378314 | 6 | 214 | 136 | 20754 | over |
| GO:0001540 | beta-amyloid binding | 0.040458 | 0.866453 | 0.00387761 | 2 | 12 | 140 | 20956 | over |
| GO:0050854 | regulation of antigen receptor-mediated signaling pathway | 0.040458 | 0.866453 | 0.00387761 | 2 | 12 | 140 | 20956 | over |
| GO:0016247 | channel regulator activity | 0.0406943 | 0.870173 | 0.00389249 | 4 | 91 | 138 | 20877 | over |
| GO:0031410 | cytoplasmic vesicle | 0.0406943 | 0.871896 | 0.00395392 | 11 | 627 | 131 | 20341 | over |
| GO:0008283 | cell proliferation | 0.0406943 | 0.871984 | 0.00395463 | 10 | 537 | 132 | 20431 | over |
| GO:0016043 | cellular component organization | 0.0419621 | 0.881182 | 0.00430786 | 26 | 2230 | 116 | 18738 | over |
| GO:0019210 | kinase inhibitor activity | 0.0422693 | 0.884251 | 0.00437893 | 3 | 46 | 139 | 20922 | over |
| GO:0051928 | positive regulation of calcium ion transport | 0.0439897 | 0.896288 | 0.00445451 | 2 | 13 | 140 | 20955 | over |
| GO:0031124 | mRNA 3'-end processing | 0.0439897 | 0.896288 | 0.00445451 | 2 | 13 | 140 | 20955 | over |
| GO:0048168 | regulation of neuronal synaptic plasticity | 0.0446911 | 0.902115 | 0.00463575 | 3 | 47 | 139 | 20921 | over |
| GO:0051649 | establishment of localization in cell | 0.0446911 | 0.902181 | 0.00463887 | 17 | 1239 | 125 | 19729 | over |
| GO:0015457 | auxiliary transport protein activity | 0.0447876 | 0.903756 | 0.00467257 | 4 | 96 | 138 | 20872 | over |
| GO:0005623 | cell | 0.0452574 | 0.907149 | 0.0048734 | 128 | 17171 | 14 | 3797 | over |
| GO:0012506 | vesicle membrane | 0.0456967 | 0.910298 | 0.00495041 | 5 | 158 | 137 | 20810 | over |
| GO:0016192 | vesicle-mediated transport | 0.0458198 | 0.911895 | 0.00502643 | 11 | 648 | 131 | 20320 | over |
| GO:0016884 | carbon-nitrogen ligase activity, with glutamine as amido-N-donor | 0.0477331 | 0.922276 | 0.00506851 | 2 | 14 | 140 | 20954 | over |
| GO:0001964 | startle response | 0.0477331 | 0.922276 | 0.00506851 | 2 | 14 | 140 | 20954 | over |
| GO:0032270 | positive regulation of cellular protein metabolic process | 0.0482187 | 0.926962 | 0.00521001 | 5 | 160 | 137 | 20808 | over |
| GO:0015276 | ligand-gated ion channel activity | 0.0482187 | 0.926962 | 0.00521001 | 5 | 160 | 137 | 20808 | over |
| GO:0022834 | ligand-gated channel activity | 0.0482187 | 0.926962 | 0.00521001 | 5 | 160 | 137 | 20808 | over |
| GO:0030136 | clathrin-coated vesicle | 0.0484277 | 0.928656 | 0.00534337 | 5 | 161 | 137 | 20807 | over |
